# Biogeographic and demographic history of the Mediterranean snakes *Malpolon monspessulanus* and *Hemorrhois hippocrepis* across the Strait of Gibraltar

**DOI:** 10.1186/s12862-021-01941-3

**Published:** 2021-11-22

**Authors:** Luis Machado, D. James Harris, Daniele Salvi

**Affiliations:** 1 CIBIO, Centro de Investigação em Biodiversidade e Recursos Genéticos, Universidade do Porto, InBIO Laboratório Associado, Vairão, Portugal; 2 Departamento de Biologia, Faculdade de Ciencias da Universidade do Porto, Porto, Portugal; 3 Institute of Evolutionary Biology (CSIC-Universitat Pompeu Fabra), Barcelona, Spain; 4 Department of Health, Life and Environmental Sciences, University of L’Aquila, L’Aquila, Italy

**Keywords:** Evolutionary history, Iberian Peninsula, Maghreb, Mediterranean Basin, North Africa, Pleistocene climatic oscillations

## Abstract

**Background:**

The contribution of North Africa to the assembly of biodiversity within the Western Palaearctic is still poorly documented. Since the Miocene, multiple biotic exchanges occurred across the Strait of Gibraltar, underlying the high biogeographic affinity between the western European and African sides of the Mediterranean basin. We investigated the biogeographic and demographic dynamics of two large Mediterranean-adapted snakes across the Strait and assess their relevance to the origin and diversity patterns of current European and North African populations.

**Results:**

We inferred phylogeographic patterns and demographic history of *M. monspessulanus* and *H. hippocrepis*, based on range-wide multilocus data, combined with fossil data and species distribution modelling, under present and past bioclimatic envelopes. For both species we identified endemic lineages in the High Atlas Mountains (Morocco) and in eastern Iberia, suggesting their persistence in Europe during the Pleistocene. One lineage is shared between North Africa and southern Iberia and likely spread from the former to the latter during the sea-level low stand of the last glacial stage. During this period *M. monspessulanus* shows a sudden demographic expansion, associated with increased habitat suitability in North Africa. Lower habitat suitability is predicted for both species during interglacial stages, with suitable areas restricted to coastal and mountain ranges of Iberia and Morocco. Compiled fossil data for *M. monspessulanus* show a continuous fossil record in Iberia at least since the Pliocene and throughout the Pleistocene.

**Conclusions:**

The previously proposed hypothesis of Pleistocene glacial extinction of both species in Europe is not supported based on genetic data, bioclimatic envelopes models, and the available fossil record. A model of range retraction to mountain refugia during arid periods and of glacial expansion (demographic and spatial) associated to an increase of Mediterranean habitats during glacial epochs emerges as a general pattern for mesic vertebrates in North Africa. Moreover, the phylogeographic pattern of *H. hippocrepis* conforms to a well-established biogeographic partition between western and eastern Maghreb.

**Supplementary Information:**

The online version contains supplementary material available at 10.1186/s12862-021-01941-3.

## Background

The Mediterranean basin is a global biodiversity hotspot and represents the major ecoregion within the Western Palearctic realm [1, 2]. The prominent role of southern Mediterranean peninsulas as Pleistocene glacial refugia and differentiation centres for Western Palaearctic thermophilic species has been corroborated by a vast amount of fossil and phylogeographic data [3, 4]. Far less is known about the occurrence and location of refugia in North Africa and their contribution to the assembly of Mediterranean biotas on both sides of the Gibraltar Strait [4–7]. Moreover, while for Europe it is well established that glacial periods represented a phase of demographic (and range) contraction for thermophilic species, with expansion phases associated with interglacial periods [3, 4, 8], less is known regarding the demographic and range dynamics of North African species during Pleistocene climatic cycles. These species might have experience favourable environmental conditions during glacial stages, as a consequence of the expansion of Mediterranean habitats [9], but this is still not well documented.

The biogeographic affinities between the European and North African portions of the Mediterranean basin are particularly accentuated across the Strait of Gibraltar, where the distance between the two regions is relatively short (~ 14 km), leading to the inclusion of Iberia and the northern part of Maghreb in the same biogeographic sub-region, the Atlantic-Mediterranean sub-centre [7, 10]. Indeed, a significant portion of the Iberian Peninsula and the Maghreb share similar climates, integrated within the Mediterranean biome, that are suitable for similar taxa in both regions [1, 7]. Since the Miocene, the complex history of intermittent connections at the Strait of Gibraltar has created opportunities for dispersal and vicariance events, that have contributed to the biotic exchange between North Africa and Western Europe [11–14]. The Messinian Salinity crisis (5.9–5.33 Ma) is often seen as a major period for biotic exchanges between Africa and Europe, due to their wide land connection, although during the Tortonian (~ 10–6 Ma), tectonic uplift and volcanism caused progressive land emergence and reduction in seaways that might have acted as filter bridges for earlier terrestrial fauna exchange [14, 15]. The opening of the Strait at the end of the Messinian Salinity crisis (5.3 million year ago, Mya) [16] established a biogeographical barrier for terrestrial organisms and isolated the Iberian and North African biotas. However, Plio-Pleistocene transmarine colonisations across the Strait have also been reported, often associated with glacial sea- level low stands [11, 17–19]. For example, in reptiles many species show shallow genetic differentiation between “European” and “African” populations, suggesting that they have independently crossed the Strait of Gibraltar, in both directions and on different occasions, throughout the Pleistocene [14, 20–22]. For many of these species the relative role of Iberia and North Africa as glacial or interglacial refugia is still unclear, as well as their role as sources of colonization toward the other side of the Strait.

Two large Mediterranean colubrid snakes, the Montpellier snake, *Malpolon monspessulanus*, and the horseshoe whip snake, *Hemorrhois hippocrepis*, present fossil and mitochondrial genetic evidence of a complex biogeographic history across the Strait of Gibraltar and suggest a North African origin of the current European populations [19]. However, the pattern of shallow divergence found in both species did not allow inference of putative refugia, or to reach a firm conclusion concerning the timing of colonization of Europe. The lack of a dense sampling and multilocus data may have prevented the identification of areas harbouring high genetic diversity and stable climatic suitability through time (putative refugia) as well as the estimate of the timing of the demographic and spatial expansions of both species across the Strait. It remains unclear whether both species became extinct in Europe during Pleistocene glaciations and only recently recolonized Europe from North Africa as suggested by Carranza et al. (2006) [19], or instead they have been present both in Europe and North Africa during the Pliocene and Pleistocene as appears conceivable based on fossil records [23–25].

Here we use an integrative approach based on an extended geographical and genetic sampling, using both mitochondrial and nuclear DNA sequence data, climate modelling techniques and fossil data to reconstruct the demographic and evolutionary history of both species and to clarify the role of North Africa and Europe as putative refuge areas and colonization source for the species during the Pleistocene. The main goal is to understand the biogeographic and evolutionary dynamics of these Mediterranean adapted species across the Strait of Gibraltar during Pleistocene climatic oscillations, and their relevance in the assembly of current European and North African biotas.

## Methods

### Dataset

**Fig. 1:**
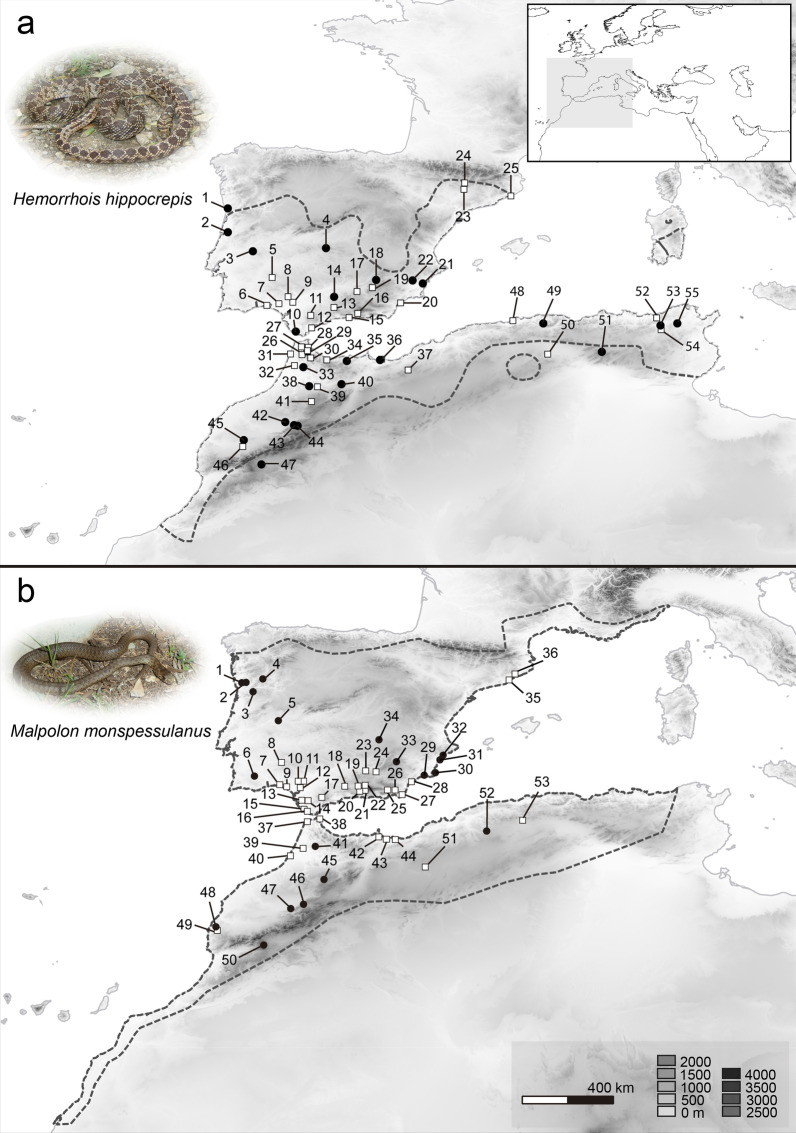
Study area and sampling of *Hemorrhois hippocrepis* (**a**) and *Malpolon monspessulanus* (**b**) (Photos by D. Salvi). Dashed lines represent species native ranges (IUCN 2008—The IUCN Red List of Threatened Species). White squares: samples used in Carranza et al. (2006) [18]; black circles: original samples from the current study

In total, we analysed 59 specimens of *Malpolon monspessulanus* and 64 specimens of *Hemorrhois hippocrepis*, covering exhaustively the distribution range of each species. The dataset comprised newly generated sequences from specimens of the collections of Centro de Investigação em Biodiversidade e Recursos Genéticos (CIBIO/InBIO) and sequences used in [19] and retrieved from GenBank. Detailed information about samples codes, sampling locality and GenBank accession numbers can be found in Fig. 1 and Additional file 1: Table S1.

### DNA extraction, amplification and sequencing

Total genomic DNA was extracted from muscle tissue, using either a standard saline method [26] or the Qiagen Dneasy^®^ Blood & Tissue extraction kit. We amplified two mitochondrial gene fragments, the cytochrome b (cyt-b; ⁓ 300 base pairs, bp) and the ribosomal 12S rRNA (12S; ⁓ 380 bp), and two nuclear gene fragments, the melanocotyin receptor 1 (MC1R; ⁓ 650 bp) and the brain-derived neurotrophic factor (BDNF; ⁓ 600 bp). These markers were successfully used in previous phylogenetic and phylogeographic studies on the same species and other Palaearctic snakes. For the cyt-b we amplified the same fragment used in [19], in order to combine our data with those from this study. Additionally, for selected samples we amplified a longer cyt-b fragments (“cyt-b long”; ⁓ 700 bp) in order to increase phylogenetic resolution. Sequenced were checked and edited in Geneious R7 [27]. Sequences of protein coding genes (cyt-b, MC1R, and BDNF) were translated into amino acids to check for the presence of stop codons. Details regarding primers and PCR protocols are provided in Table 1.

**Table 1:** Details on gene amplifications: primers’ name, sequence and references, and PCR conditions used

Gene	Primer	Primer sequence	References	PCR conditions (°C(time) × number of cycles)
cyt-b	CB1	5′ CCATCCAACATCTCAGCATGATGAAA 3′	[28]	94° (3′), [94° (30″), 50° (45″), 72° (2′) × 35], 72° (10′)
	CB2	5′ CCCTCAGAATGATATTTGTCCTCA 3′	[28]	
	MVZ 16	5′ AAATAGGAAGTATCACTCTGGTTT 3′	[29]	
12S	12Sa	5′ CTGGGATTAGATACCCCACTAT 3′	[30]	94° (3′), [94° (30″), 49° (30″), 72° (1′) × 35], 72° (5′)
	12Sb	5′ GAGGGTGACGGGGCGGTGTGT 3′	[30]	
MC1R	MC1R F	5′ GGCNGCCATYGTCAAGAACCGGAACC 3′	[31]	94° (3′), [94° (30″), 50° (30″), 72° (1′) × 35], 72° (5′)
	MC1R R	5′ CTCCGRAAGGCRTAGATGATGGGGTCCAC 3′		
BDNF	BDNF_DRV_F	5′ ACCATCCTTTTCCTKACTATGG 3′	[32]	94° (3′), [94º(30″), 50° (45″), 72° (2′) × 35], 72° (10′)
	BDNF_DRV_R	5′ CTATCTTCCCCTTTTAATGGTC 3′		

### Phylogeographic analysis

Multiple sequence alignments were performed using MAFFT 7 [33] with default settings, except for the 12S alignment, for which we used the Q-INS-i-strategy that takes in to account the secondary structure of the RNA. Haplotype reconstruction for nuclear gene fragments was performed in PHASE 2.1 [34] as implemented in DNAsp 5 [35]. Haplotype (*h*) and nucleotide diversity (π) were also calculated in DNAsp for the concatenate mtDNA alignment (cyt-b and 12S) and each nuclear marker (phased sequences). Phylogenetic relationships between haplotypes were inferred using the parsimony network approach implemented in TCS 1.21 with a 95% connection limit [36]. Network approaches are the most appropriate for intraspecific gene evolution, particularly when few characters for phylogenetic analysis are available due to shallow levels of divergence. Haplotype networks were inferred for each gene alignment and for the concatenated mtDNA alignment.

The demographic histories of both snakes were inferred using the Extended Bayesian Skyline Plots (EBSP) implemented in BEAST 1.8.0 [37]. EBSP is a coalescent based method that estimates current and past effective population sizes (Ne) from genetic data from multiple independent loci, which significantly improves the power of detecting past demographic fluctuations and reduces estimation errors [37, 38]. EBSP were performed with the concatenated mtDNA dataset, plus the two nuclear gene datasets, implementing the best-fit models of nucleotide substitution selected by jModelTest 2.1.10 [39] (Additional file 1: Table S2). In order to estimate the time of demographic events within *Malpolon monspessulanus* and *Hemorrhois hippocrepis* we used the rate of evolution of 1.3% substitution/million years (s/my) estimated for mitochondrial DNA in colubrids by Daza, Smith, Páez, & Parkinson (2009) [40]. We defined a lognormal distribution on the ucld.mean parameter with a mean of 0.013 substitutions/my and a 95% confidence interval of [0.009–0.0179], thereby covering substitution rates estimated in previous studies on colubrids [41]. BEAST analyses were run twice for 150 million generations sampling every 5000 steps, (burn-in = 10%); run convergence was assessed using Tracer 1.5 [42] and EBSP were drawn using the package *ggplot* [43].

### Predictive modelling

We evaluated the suitability of Iberian and North African environments for *Malpolon monspessulanus* and *Hemorrhois hippocrepis* under present, Holocene (6 thousand years ago, kya), Last Glacial Maximum (LGM; 23–18 kya) and Last Interglacial (LIG; 140–120 kya) bioclimatic envelopes, using the maximum-entropy algorithm implemented in MaxEnt 3.3.3e [44]. This presence-only modelling technique fits with the elusive nature of snakes and has a good performance with both small and large sample sizes [45]. To prepare the environmental layers we used bioclimatic data from WorldClim version 1.4 [46]. We downloaded 19 bioclimatic variables available for current and past conditions at a resolution of 5 arc-min ~ 10 km. We cut each bioclimatic raster to a study area that accommodates both species native distribution and putative range shifts across Pleistocene: Xmin = − 17.65, Ymin = 14.69, Xmax = 27.60, and Ymax = 49.1. Among all bioclimatic variables, we selected six variables that have high biological significance for snakes [47, 48] and show a Spearman's correlation lower than 0.77: bio6, Minimum Temperature of Warmest Month, bio7, Temperature Annual Range; bio 8, Mean Temperature of Wettest Quarter; bio9, Mean Temperature of Driest Quarter; bio 15, Precipitation Seasonality; bio16, Precipitation of Wettest Quarter. Using different sets of not-autocorrelated variables gave consistent results, thus suggesting that the models are robust to variable selection. In order to project suitability of the species in the past, we downloaded the same bioclimatic variables for LIG, LGM and Holocene periods from the CCSM4 (CC) Global Climate Model as well as from the MIROC-ESM (MIROC) and MPI-ESM-P (MPI) models for LGM and Holocene.

We collected 10,792 presence data points for the two species (*M. monspessulanus*: 7489; *H. hippocrepis*: 3303) from field observations [49], the GBIF [50], atlas of moroccan herpetofauna [49, 51] and from the collections of the Centre d’Ecologie Fonctionelle et Evolutive (CEFE, Montpellier), the CIBIO/InBIO, the Muséum National d’Histoire Naturelle (MNHN, Paris) and the Natural History Museum (NHM, London). To account for sampling bias, first we filtered occurrence data in order to have only one record in each 10 km square (corresponding to the resolution of bioclimate variables). Second, we filtered the occurrence dataset taking in to account the bioclimatic data along the study area. We performed a principal component analysis (PCA) using the values of the six bioclimatic variables at each locality with a presence record. Based on the PCA result, we realized a K-means cluster analysis to group each of these localities according to four different bioclimatic clusters. Having assign a bioclimatic cluster, we filter the presence data in order to maximize bioclimatic variance. All this analysis was performed in R with packages *factoextra* and *ggplot2* [52–54]. The final dataset of occurrence records included a total of 325 records of *H. hippocrepis* and 367 records of *M. monspessulanus* (Additional file 1: Tables S3 and S4 and Fig. S1 for geographic details)*.* In order to evaluate the degree of spatial autocorrelation we calculated the Nearest neighbour index (NNI) in QGIS 2.8.3 [76] for *H. hippocrepis* (NNI = 0.59; Z-score = − 14.25) and *M. monspessulanus* (NNI = 0.65; Z-score = − 12.62), that revealed a low degree of clustering in the distribution of records.

MaxEnt analyses were run with random selection of 70% presence records; the remaining 30% were used as test data on 25 replicates models. The importance of each variable for model building was assessed with jackknife analyses. Curve plots with a relationship between species presence and bioclimatic variables values were obtained. Models were also tested with received-operated characters (ROC) for the agreement between observed species presence and projected distribution. The area under the curve (AUC) of the ROC analysis provide a measure of the model performance, ranges from 0.5 (randomness) to 1 (perfect discrimination). A good model accuracy is considered with an AUC ≥ 0.7. All projections produced by MaxEnt were posteriorly edited in QGIS.

### Fossil record

In order to obtain insights on the occurrence of both species in Iberia and North Africa during the last million years, we gathered fossil data for both species from the Fossil Fishes, Amphibians, Reptiles, Birds database, fosFARbase [55]. For both regions, we also retrieved a few records identified as *Malpolon* sp. or *Coluber* sp. (*hippocrepis* was originally included within *Coluber* Linnaeus, 1758) for which their attribution to either of the two study species cannot be ruled out (Hugues-Alexandre Blain, personal communication). These latter records were treated separately from those identified at the specie level.

## Results

### Phylogeographic analysis

For most of the collected samples (Fig. 1) we were able to obtain sequences of the short cyt-b fragment and of nuclear markers, whereas amplification rate was lower for the long cytb-fragment and for the 12S (Additional file 1: Table S1), likely because of the poor preservation of specimens, many of which were road-killed animals.

**Fig. 2:**
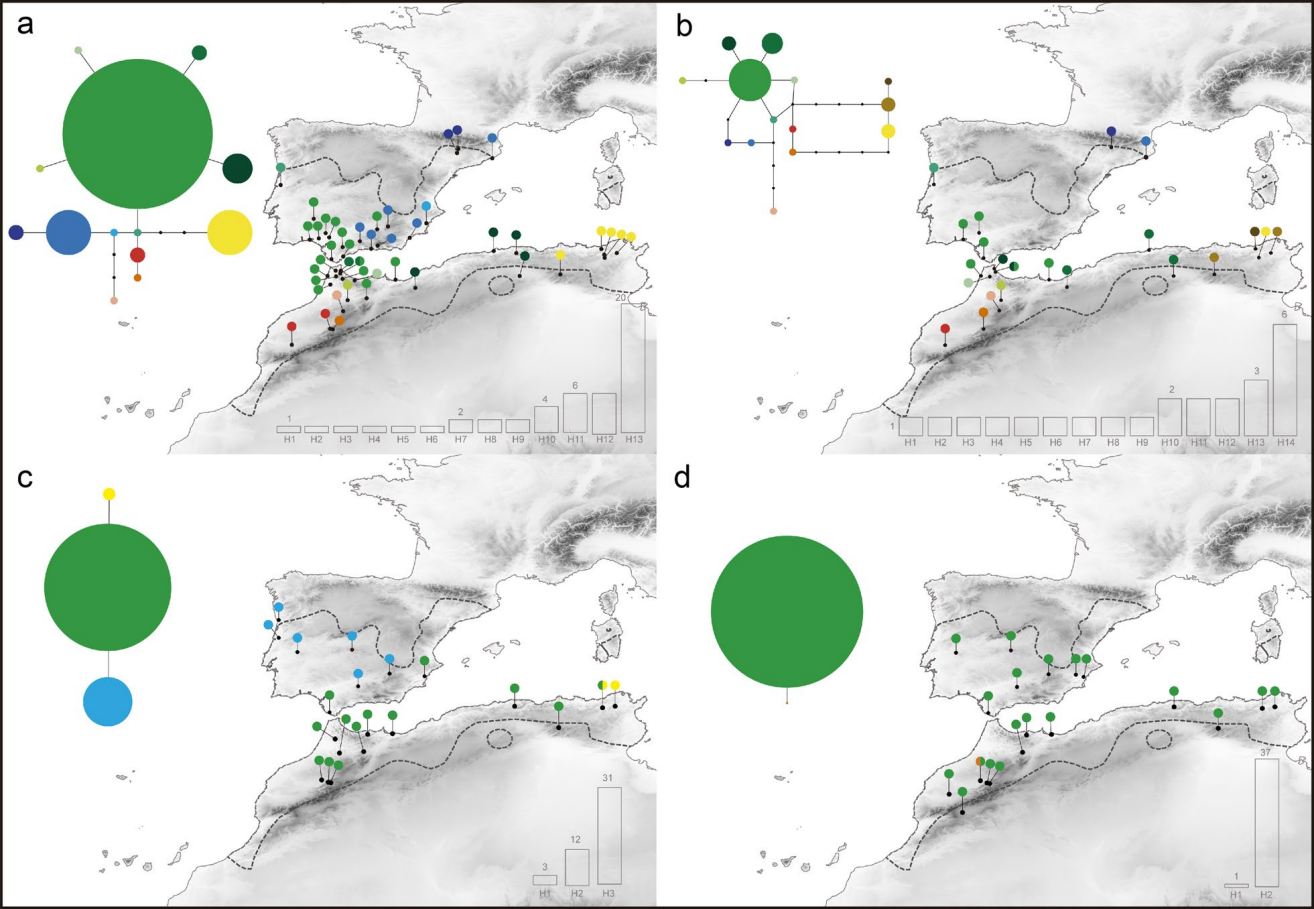
Statistical parsimony networks recovered in *Hemorrhois hippocrepis*: **a** cyt-b haplotype network; **b** mtDNA (cyt-b + 12S) haplotype network; **c** BDNF haplotype network; **d** MC1R haplotype network

**Fig. 3:**
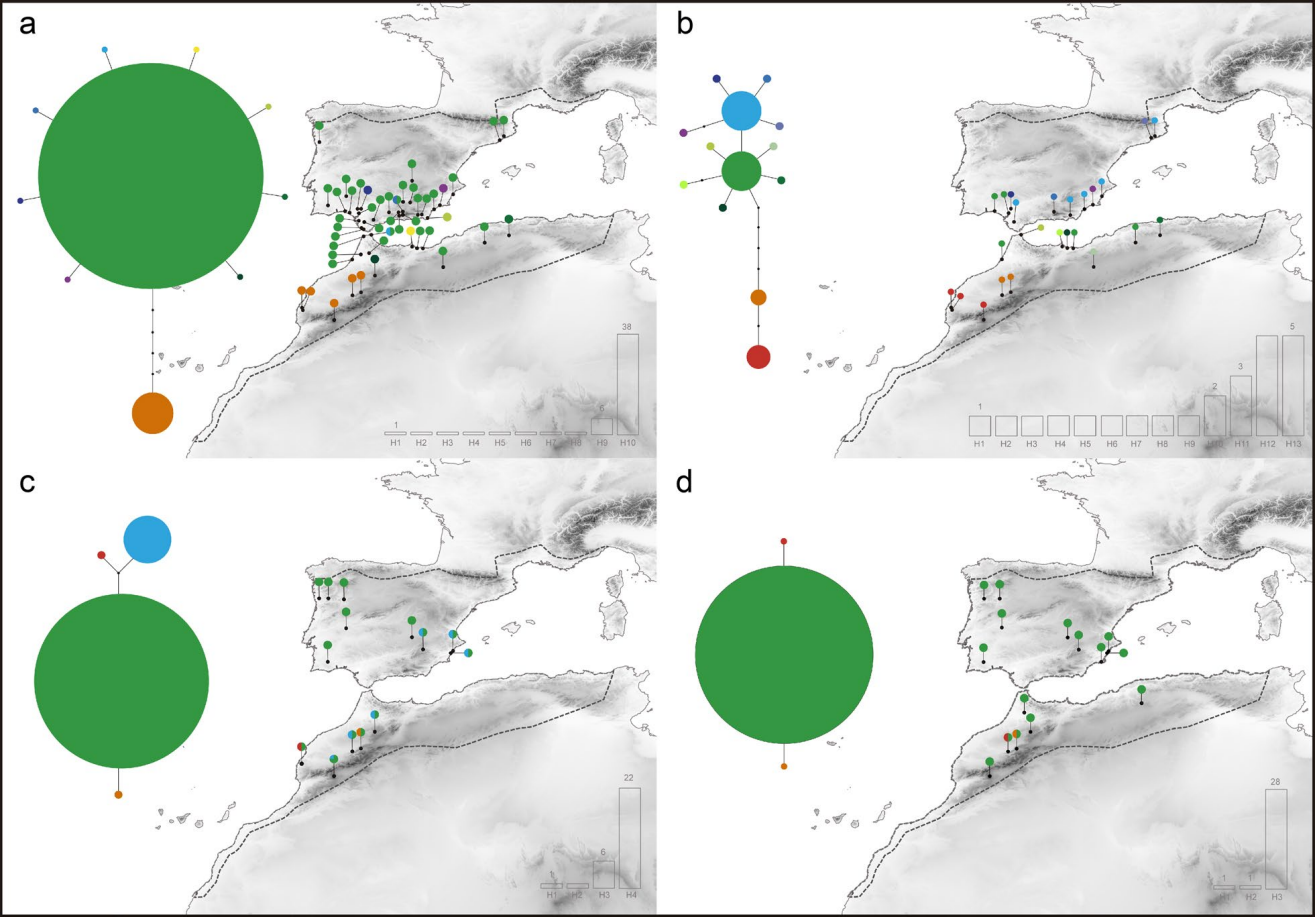
Statistical parsimony networks recovered in *Malpolon monspessulanus*: **a** cyt-b haplotype network; **b** mtDNA (cyt-b + 12S) haplotype network; **c** BDNF haplotype network; **d** MC1R haplotype network

Both species showed high mtDNA haplotype diversity and low nucleotide diversity (*Mm*, *h* = 0.913 ± 0.036, π = 0.0054 ± 0.00091; *Hh*, *h* = 0.924 ± 0.038, π = 0.006 ± 0.00086). Diversity estimates were significantly lower at nuclear genes (MC1R: *Mm*, *h* = 0.131 ± 0.082, π = 0.00022 ± 0.00014; *Hh*, *h* = 0.053 ± 0.04, π = 0.00009 ± 0.00009; BDNF: *Mm*, h = 0.434 ± 0.0095, π = 0.00156 ± 0.00035; *Hh*, *h* = 0.484 ± 0.065, π = 0.00092 ± 0.00015). Haplotype diversity at mitochondrial and nuclear genes is higher within North Africa in both species (Figs. 2, 3; Additional file 1: Fig. S2, 3).

For both *H. hippocrepis* and *M. monspessulanus,* the cyt-b and the concatenated mtDNA alignments were resolved into a single statistical parsimony network (Figs. 2a, b, 3a, b; Additional file 1: Fig. S2, 3)*.* In the cyt-b network of both species we found one haplotype with high frequency, representing 42% and 73% of the samples for *H. hippocrepis* and *M. monspessulanus*, respectively. Singletons account for 6 out of 13 haplotypes for *H. hippocrepis* and 8 out of 10 haplotypes for *M. monspessulanus* (Figs. 2a, 3a). For the concatenated mtDNA alignments the most common haplotype found in *H. hippocrepis* represents 25% of the samples, whereas two equally common haplotype represent each 21% of *M. monspessulanus* samples. Singletons account for 9 out of 14 haplotypes for *H. hippocrepis* and 9 out of 13 haplotypes for *M. monspessulanus* (Figs. 2b, 3b)*.* Also, BDNF and MC1R networks show a highly frequent haplotype, representing 67% of the samples for BDNF and 97% for MC1R in *H. hippocrepis,* and 73% of the samples in BDNF and 93% for MC1R in *M. monspessulanus* (Figs. 2c, d, 3c, d)*.*

The phylogeographic pattern of both species shows exclusive mtDNA haplogroups in Iberia (Iberian haplogroup, blue colours; Figs. 2a, b, 3a, b; Additional file 1: Fig. S2, 3) and the High Atlas Mountains of Morocco (red–orange colours), whereas one haplogroup was shared across the Strait of Gibraltar (Ibero-Maghrebian haplogroup, green colours), with the most frequent haplotype being distributed in northern Morocco and southern Iberia. In *H. hippocrepis* one additional divergent haplogroup is found in eastern Algeria and Tunisia (yellow–brown colours). The number of exclusive mitochondrial haplotypes is slightly higher in North Africa than in the Iberian Peninsula for both *H. hippocrepis* and *M. monspessulanus.* Nuclear haplotype networks show a few haplotypes with a shallow phylogeographic structure. Exclusive nuclear haplotype, in both markers, are only present in North Africa and concentrated in mountain regions (Figs. 2, 3).

**Fig. 4:**
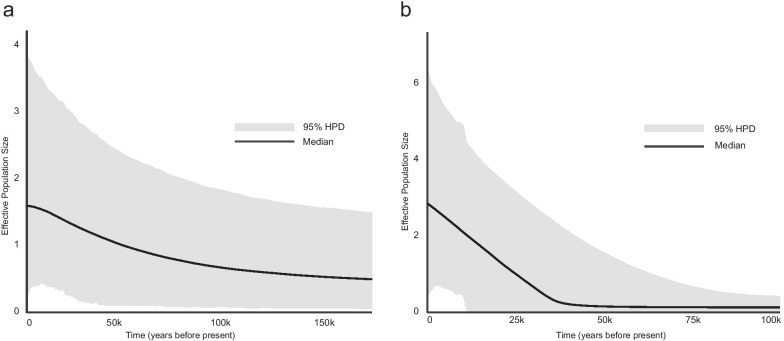
Extended Bayesian Skyline Plots of the historical demographic reconstruction: **a** *Hemorrhois hippocrepis* and **b** *Malpolon monspessulanus*

The historical demographic reconstructions based on mitochondrial and nuclear loci show a pattern of demographic expansion for both species during the last glacial period, that is particularly marked in *M. monspessulanus* with a sudden increase at ⁓ 40 kya (Fig. 4). For both species, a single demographic change obtained the highest posterior probability density.

### Predictive modelling

**Fig. 5:**
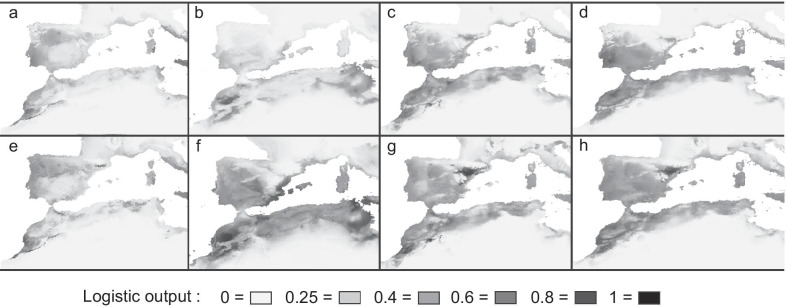
Species distribution models (SDMs) of (**a**–**d**) *Hemorrhois hippocrepis* and (**e**–**h**) *Malpolon monspessulanus* for (**a**, **e**) Last Interglacial (LIG), (**b**, **f**) Last Glacial Maximum (LGM), (**c**, **g**) Holocene, and (**d**, **h**) Present conditions. For LGM and Holocene the SDM is based on the MIROC circulation model (see Additional file 1: Fig. S4 for SDMs based on the CC and MPI circulation models)

Species distribution models for *H. hippocrepis* and *M. monspessulanus* recovered AUC values of 0.96 ± 0.02 and 0.94 ± 0.02, respectively, indicating good performance of the models (Additional file 1: Table S5). For both snakes the Precipitation of Wettest Quarter (bio16) was the most informative variable, followed by the Mean Temperature of Wettest Quarter (bio8) (Additional file 1: Table S5). The present‐day habitat suitability reconstructions showed continuous high suitability across regions with Mediterranean climates, which fits well with the known species distributions (Fig. 5d, h). A lower fit is apparent for *M. monspessulanus* in the northernmost region of Iberia, characterised by oceanic climate, where the species is currently absent (see the species range in Fig. 1b), but the model shows some suitability (Fig. 5h). In both snakes, projections during LIG showed a lower habitat suitability in North Africa, with a fragmentation of suitable areas that were limited to coastal and mountain areas of Morocco, and with two isolated areas in western and eastern Maghreb (Fig. 5a, e). On the other hand, models predict an increase of suitable areas during the LGM, especially in the Maghreb (Fig. 5b, f), which was particularly evident for *M. monspessulanus.* Habitat suitability models at the LGM show similar patterns among the three projections in both species (MIROC, MPI and CCSM; Fig. 5b, f and Additional file 1: Fig. S4). Projections for Holocene recover areas of suitability for both snakes similar to present conditions in all models (Fig. 5c, g and Additional file 1: Fig. S4).

### Fossil record

**Fig. 6:**
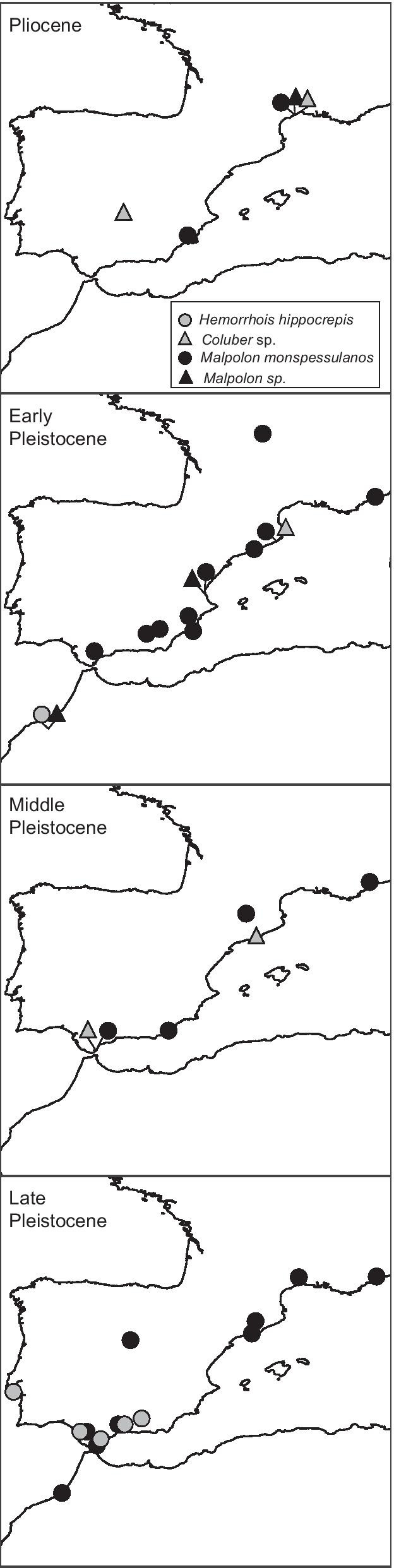
Fossil data available for *Hemorrhois hippocrepis* (grey circles) and *Malpolon monspessulanus* (dark circles) in the Pliocene, Early Pleistocene, Middle Pleistocene, and Late Pleistocene

Compiled fossil data for *M. monspessulanus* show a continuous fossil record in Iberia since the Pliocene and throughout the Pleistocene (Fig. 6; Additional file 1: Tables S6, 7). The oldest possible fossil record for this species may be either from the Early Pliocene (4.9–5 Mya) of Puerto de la Cadena (Spain) (but this record is based on a few vertebrae) or the Late Pliocene (3.5–3.6 Mya), and 14 records from Iberia are referred to the Early Pleistocene (0.8–2.6 Mya). The fossil record of *H. hippocrepis* is poor with the oldest remains assigned to this species found in the Early Pleistocene (2.5 Mya) of Ahl al Oughlam (Morocco) and seven records from the Late Pleistocene from Iberia (0.127–0.01 Mya). All other records from Pliocene to Middle-Pleistocene (0.13–3.6 Mya) come from Iberia and are identified at the genus level.

## Discussion

The integrative approach used in this study, combining multi-locus genetic data, fossil records and climate suitability models, provided new insights into the evolutionary and demographic history of the snakes *Hemorrhois hippocrepis* and *Malpolon monspessulanus*, with implications on the biotic exchanges across the Strait of Gibraltar during Quaternary glaciations. Results support a pattern of demographic and range contraction in North Africa during interglacial epochs followed by glacial expansion, which is unusual across Mediterranean thermophilic organisms. Contrary to the previous hypothesis of glacial extinction for both species in Iberia [19], evidence supports a long history of the two species in the Iberian Peninsula with persistence during Pleistocene climatic oscillations. Finally, denser sampling in North Africa and longer gene fragments allowed identification of areas that during the Pleistocene served either as refugia or as source for the dispersal towards the southern Iberian Peninsula.

### Glacial expansion for the two Mediterranean snakes during the Pleistocene

Historical demographic reconstructions indicate that both species underwent a demographic expansion during the Late Pleistocene (Fig. 4). According to our molecular calibration, the demographic expansion started around 40 kya in *M. monspessulanus* and sometime before in *H. hippocrepis*, a timeframe that coincides with the last glacial period. This finding is not consistent with demographic and phylogeographic patterns observed in Europe. In most temperate and Mediterranean species studied to date, range-wide patterns of genetic variation have been explained by phases of range and demographic contraction associated to unfavourable glacial climates, while expansion phases coincided with more suitable interglacial conditions [3, 56–59]. In the last decade, exceptions to this general ‘Expansion–Contraction’ model have been described, especially for coastal and insular species where demographic and range expansions have been associated to the glacial increase of climatically suitable coastal lowlands [60–63]. While far less case studies are available for North Africa, for mesic vertebrates such as *H. hippocrepis* and *M. monspessulanus*, that is, species adapted to arid conditions but still requiring some moisture, a model of expansion and contraction was proposed by Brito et al. (2014) [9], following the cycles of wet and arid phases in the area. According to Brito et al. (2014) [9], mesic species would expand their ranges under wet conditions associated with the Sahara Desert contraction, whereas, during unfavourable arid periods, mountain areas would act as refuge for these species by preserving mesic habitats. These predictions are in line with the results of phylogeographic, demographic and modelling analyses on *H. hippocrepis* and *M. monspessulanus*, suggesting a close association between the demographic and spatial expansion of these snakes with the expansion of Mediterranean habitats in North Africa during the last glacial period. Species distribution models indicate that in this region the amount of climatically suitable areas available to *H. hippocrepis* and *M. monspessulanus* was substantially higher during the last glacial period compared to the interglacials (Fig. 5 and Additional file 1: Fig. S4). The historical demographic reconstructions show that in both species a sudden demographic expansion coincided with favourable conditions in the Maghreb during the last glacial period, slightly earlier in *H. hippocrepis* than in *M. monspessulanus*. These snakes also show the highest haplotype diversity, including divergent and private haplotypes, in proximity of the High Atlas and Algerian mountains (Figs. 2, 3 and Additional file 1: Fig. S2–3) suggesting a key role of mountain areas of North Africa as long term refugia during Pleistocene climate oscillations. A remarkably similar pattern was found in a recent study on the North African snake *Daboia mauritanica* [47], that inferred range contractions and allopatric diversification during warmer interglacial periods and range expansion during colder glacial periods. Therefore, mounting evidence supports a demographic model for Mediterranean species in North Africa with allopatric isolation following retraction in mountain refugia during arid periods, and (demographic and spatial) expansion associated to an increase of Mediterranean habitats during the last glacial epoch. In this regard, it must be noted that the history of spatial and temporal climatic variability of North Africa is complex and still debated, and that a strict association of glacial stages with wet conditions, and of interglacial stages with arid conditions, is too simplistic [64–66]. Palaeoclimatic records show several wet periods in North Africa during the last glacial period, supporting a scenario of expansion of Mediterranean habitats, but with at least one period of aridification at ⁓ 60 kya [64, 65, 67]. On the other hand, the last interglacial might have been a short humid period [66], with arid conditions being prevalent just before it, from ⁓ 130 to ⁓ 170 kya [64]. Further phylogeographical and palaeoclimatic data are necessary to understand, on a finer time scale, the history of the North African biota during Pleistocene climatic oscillations.

### Recent colonization or long-term persistence in the Iberian Peninsula?

A previous study based on mtDNA data recover a phylogeographic pattern of shallow divergence across the range of *H. hippocrepis* and *M. monspessulanus*, especially in the Iberian Peninsula [19]. This is unusual among Mediterranean taxa that usually show deep and complex phylogeographic structure in this region resulting from multiple isolated refugia during Pleistocene Ice Ages [68]. Such an unusual pattern of low genetic diversity in Iberia and limited differentiation between Maghreb and Iberian populations was explained as a very recent arrival (Late Pleistocene) in the Iberian Peninsula of these two snakes. According to this scenario, fossils of *H. hippocrepis* and *M. monspessulanus* from the Pliocene of Iberia derived from ancient populations of these species that underwent extinction during earlier glaciation events [19].

Based on the results of this study we can assess two additional hypotheses. First, the low mitochondrial diversity observed in the previous study in these two species could be explained by a selective process acting on the mitochondrial genome (i.e., ‘mitochondrial sweep’) [69]. A ‘mitochondrial sweep’ was suggested for two geckos to explain the shallow mitochondrial diversity observed in Europe as compared to higher nuclear polymorphisms observed across their range [70]. However, the nuclear data obtained in this study do not support this hypothesis for *H. hippocrepis* and *M. monspessulanus*, as we found very limited genetic diversity also in two nuclear genes. In this regard, while a low rate of evolution may explain the low diversity observed at the BDNF locus, the MC1R locus usually show high polymorphism and phylogenetic structure in many North African and European reptiles [70–75].

A second hypothesis is that one or more lineages endemic to Iberia were overlooked in the previous study, and these would provide evidence for the long-term persistence during Plio- Pleistocene glaciations of both species. Our results strongly support this hypothesis of a long history of the two species in Iberian Peninsula. For both species we unveiled two main haplogroups in Iberia, one shared with Northwest Africa (Ibero-Maghrebian haplogroup), and another one endemic to eastern and northern Iberia (Iberian haplogroup). The latter testify that at least part of the current species’ diversity in Europe evolved in situ and persisted during Pleistocene climatic oscillations. This is particularly evident in analyses based on longer gene fragments (Additional file 1: Fig. S2-3), suggesting that these endemic Iberian lineages may have been undetected in the previous study [19] because of a lack of phylogeographic resolution of the short gene fragment used. The long-term persistence in Europe of the two snakes is also in line with fossil data. *Malpolon monspessulanus* show an extensive fossil record (in many cases represented by several skeletal elements) in Iberia since the Pliocene (4.9–5 Mya) and the Early Pleistocene (1–2.6 Mya), and throughout Middle and Late Pleistocene [25, 76–78] (Fig. 6; Additional file 1: Table S7). For *H. hippocrepis* the fossil record is less rich and unfortunately older records from Iberia are only identified at the genus level. Finally, species distribution models indicate suitable areas in Iberia during last glacial period for both species, particularly in southeast Iberia, which are coincident with the occurrence of Iberian endemic haplogroups and with the majority of fossil remains (Figs. 2, 3, 5, 6), thus providing solid arguments against the hypothesis of a glacial extinction of both species in Europe [19].

While the endemic Iberian haplogroups indicate the persistence of these two species in Europe during Pleistocene glaciations, the Ibero-Maghrebian haplogroups found in southern Iberian and Northwest African populations of both species suggest recent gene flow across the Strait of Gibraltar. In line with the finding of Carranza et al. (2006) [19], the higher diversity of these Ibero-Maghrebian haplogroups is found in Morocco, supporting a recent colonization of the Iberian Peninsula from Northwest Africa. Trans-marine dispersal of these two species was likely facilitated by the reduced extent of the sea channel (despite a possibly faster marine flow) at the Strait of Gibraltar during sea-level low stands associated with glacial stages [11, 79]. Indeed, overseas dispersion across the Strait of Gibraltar have been inferred for several terrestrial organisms throughout the Pleistocene (see [14] for a review). According to our historic demographic reconstruction, during the last glacial stage a demographic expansion took place in both species that could have been associated with their colonization of southern Iberia across the Strait. Interestingly, in both species the observed phylogeographic pattern suggest that these Maghrebian lineages, after their arrival in Iberia, established a wide area of admixture with endemic Iberian lineages in the southern and south-eastern region.

To answer the main question on the biogeographic origin of the European populations of these two snakes, this study shows that the phylogeographic pattern of both species is more complex than previously thought and suggests that both long-term persistence in the east and north Iberia and recent colonization of south Iberia from North Africa have contributed to the current diversity of these two snakes within the Iberian Peninsula.

### Insights into North African phylogeography

The inventory of phylogeographic patterns in North Africa is far from complete [7], however as more and more studies accumulate, some general biogeographic patterns start to emerge, suggesting that the genetic diversity of many Maghrebian taxa have been shaped by the same biogeographic events [80–83].

The major biogeographic break between western and eastern Maghreb, documented in different organisms (see [83] for a recent review), is found also in *H. hippocrepis* corresponding to the Kabylian region (central-eastern Algeria). The climatic suitability model for this species during the LIG shows a clear fragmentation between the western and eastern ranges, with low suitability across central Algeria (Fig. 5). During this period suitable areas in proximity of the Tell and Aurès mountains could have acted as an isolated eastern refuge for the species (Fig. 5).

Both *H. hippocrepis* and *M. monspessulanu*s show two main lineages in the western Maghreb, one in northern regions from Morocco to central Algeria (Figs. 2, 3; haplogroups with green tones), and another one restricted to the High Atlas (Figs. 2, 3; haplogroups with orange and red tones). This latter lineage, endemic to southern mountains, was not detected by Carranza et al. (2006) [19] because of a lack of sampling in this region. This phylogeographic pattern suggests the occurrence of two isolated refugia in the western Maghreb during unfavourable period of the Pleistocene: one in northern Morocco, and another one in the High Atlas Mountains. The climatic suitability model for both species during the LIG support the occurrence of both refugia (Fig. 5).

## Conclusions

In this study the use of denser sampling, longer gene fragments, multilocus analyses and species distribution modelling allowed the reconstruction of a more complex scenario for the evolutionary and demographic history of *H. hippocrepis and M. monspessulanus* across the Strait of Gibraltar compared to previous studies. The hypothesis of Pleistocene glacial extinction of both species in Europe is not supported based on increased genetic data and a detailed analysis of the available fossil record. Instead, results of phylogeographic, demographic and modelling analyses suggest that, while during the last glacial stage one lineage of both species re-colonised the southern Iberian Peninsula from North Africa, multiple populations of these two snakes survived in northern and eastern refugia in the Iberian Peninsula throughout Pleistocene Ice Ages. A model of glacial expansion (demographic and spatial) associated to an increase of Mediterranean habitats during the last glacial epoch, and of retraction in mountain refugia during arid phases, emerges as a general pattern for mesic vertebrates in North Africa [9, 47]. Finally, the phylogeographic pattern of *H. hippocrepis* conforms with a well-established biogeographic partition between western and eastern Maghreb [83].

## Supplementary Information

**Additional file 1:** **Table S1**: Details on samples’ location, gene fragments sequenced and GenBank accession numbers of sequences, for *H. hippocrepis* and *M. monspessulanus*. **Table S2**: Summary statistics and substitution models used for the Extended Bayesian Skyline Plot analyses. **Table S3**: Occurrence data for *H. hippocrepis* used as input for Maxent modelling analysis. **Table S4**: Occurrence data for *M. monspessulanus* used as input for Maxent modelling analysis. **Table S5**: Summary statistics of Maxent performance, each bioclimatic variable contribution (%) and output (logistic threshold for the “10th percentile”, “Equal sensitivity and specificity” and “Maximum sensitivity plus specificity”) for models of *H. hippocrepis* and *M. monspessulanus*. **Table S6:** Fossil data of *Hemorrhois hippocrepis* (source: fosFARbase, Bohme & Ilg, 2003). Mya: million years ago; epoch boundaries follow Gradstein et al. (2005). **Table S7**: Fossil data of *Malpolon monspessulanus* (source: fosFARbase, Bohme & Ilg, 2003). Mya: million years ago; epoch boundaries follow Gradstein et al. (2005). **Fig. S1:** Occurrence points used for Maxent climate modeling: (a) *H. hippocrepis*; (b) *M. monspessulanus*. Dashed lines represent species native distribution. **Fig. S2**: Haplotype network recovered in *H. hippocrepis* using the 700 bp cyt-b fragment. **Fig. S3**: Haplotype network recovered in *M. monspessulanus* using the 700 bp cyt-b fragment. **Fig. S4**: Species distribution models (SDMs) of *H. hippocrepis* and *M. monspessulanus* for Holocene and Last Glacial Maximum (LGM) conditions based on the MPI and CC circulation models.

## Data Availability

All genetic data in this study, either original or from previous studies, is available on GenBank (www.ncbi.nlm.nih.gov/genbank/), with respective accession numbers listed in Additional file 1: Table S1. Presence data for *Hemorrhois hippocrepis* and *Malpolon monspessulanus* used as input in Maxent is available in Additional file 1: Table S3–4. Bioclimatic data used in Maxent was retrieved from http://www.worldclim.com/version1. Fossil Data for both species was retrieved from www.wahrestaerke.com
