# Supplementary material for: Biogeographic and demographic history of the Mediterranean snakes Malpolon monspessulanus and Hemorrhois hippocrepis across the Strait of Gibraltar
Source: BMC Ecol Evol. 2021 Nov 22;21:210. doi: 10.1186/s12862-021-01941-3 (PMC8609814; doi:10.1186/s12862-021-01941-3)
Supplement: Supplementary file 1 — Additional file 1: Table S1: Details on samples’ location, gene fragments sequenced and GenBank accession numbers of sequences, for H. hippocrepis and M. monspessulanus. Table S2: Summary statistics and substitution models used for the Extended Bayesian Skyline Plot analyses. Table S3: Occurrence data for H. hippocrepis used as input for Maxent modelling analysis. Table S4: Occurrence data for M. monspessulanus used as input for Maxent modelling analysis. Table S5: Summary statistics of Maxent performance, each bioclimatic variable contribution (%) and output (logistic threshold for the “10th percentile”, “Equal sensitivity and specificity” and “Maximum sensitivity plus specificity”) for models of H. hippocrepis and M. monspessulanus. Table S6: Fossil data of Hemorrhois hippocrepis (source: fosFARbase, Bohme & Ilg, 2003). Mya: million years ago; epoch boundaries follow Gradstein et al. (2005). Table S7: Fossil data of Malpolon monspessulanus (source: fosFARbase, Bohme & Ilg, 2003). Mya: million years ago; epoch boundaries follow Gradstein et al. (2005). Fig. S1: Occurrence points used for Maxent climate modeling: (a) H. hippocrepis; (b) M. monspessulanus. Dashed lines represent species native distribution. Fig. S2: Haplotype network recovered in H. hippocrepis using the 700 bp cyt-b fragment. Fig. S3: Haplotype network recovered in M. monspessulanus using the 700 bp cyt-b fragment. Fig. S4: Species distribution models (SDMs) of H. hippocrepis and M. monspessulanus for Holocene and Last Glacial Maximum (LGM) conditions based on the MPI and CC circulation models. [file 12862_2021_1941_MOESM1_ESM.pdf]

Additional File 1 - Supplementary Material for:

Biogeographic and demographic history of the Mediterranean snakes *Malpolon monspessulanus* and *Hemorrhois hippocrepis* across the Strait of Gibraltar

Luis Machado, D. James Harris, Daniele Salvi*

* Corresponding author: Daniele Salvi, danielesalvi.bio@gmail.com

Supplementary Tables.

Table S1 - Details on samples' location, gene fragments sequenced and Genbank accession numbers of sequences, for *H. hippocrepis* and *M. monspessulanus*……………………………………………………………………2

Table S2 - Summary statistics and substitution models used for the Extended Bayesian Skyline Plot analyses…….7

Table S3: Occurrence data for *H. hippocrepis* used as input for Maxent modelling analysis………………………..8

Table S4 - Occurrence data for *M. monspessulanus* used as input for Maxent modelling analysis…………………..14

Table S5 - Summary statistics of Maxent performance, each bioclimatic variable contribution (%) and output (logistic threshold for the "10th percentile", "Equal sensitivity and specificity" and "Maximum sensitivity plus specificity") for models of *H. hippocrepis* and *M. monspessulanus*……………………………………………………21

Table S6 - Fossil data of *Hemorrhois hippocrepis* (source: fosFARbase, Bohme & Ilg, 2003). Mya: million years ago; epoch boundaries follow Gradstein et al (2005)…………………………………………………………...22

Table S7 - Fossil data of *Malpolon monspessulanus* (source: fosFARbase, Bohme & Ilg, 2003). Mya: million years ago; epoch boundaries follow Gradstein et al (2005)……………………………………………………………..23

Supplementary Figures.

Figure S1: Occurrence points used for Maxent climate modeling: a) *H. hippocrepis*; b) *M. monspessulanus*. Dashed lines represent species native distribution……………………………………………………………………25

Figure S2: Haplotype network recovered in *H. hippocrepis* using the 700 bp cyt-b fragment……………………26

Figure S3: Haplotype network recovered in *M. monspessulanus* using the 700 bp cyt-b fragment…………………26

Figure S4: Species distribution models (SDMs) of *H. hippocrepis* and *M. monspessulanus* for Holocene and Last Glacial Maximum (LGM) conditions based on the MPI and CC circulation models………………………………..27

Supplementary Tables

Table S1 - Details on samples' location, gene fragments sequenced and Genbank accession number of sequences, for *H. hippocrepis* and *M. monspessulanus*.

Species	Locality Code	Country – Region/District	Sample Code	Latitude / Longitude	cyt-b (short)	cyt-b (long)	12S	MC1R	BDNF	Reference
H. hippocrepis	1	Portugal – Porto	DB4299	41.147 / -8.579	-	-	-	-	OL547558	This study
H. hippocrepis	2	Portugal – Coimbra	DB13782	40.194 / -8.579	-	OL547488	OL547509	-	OL547559	This study
H. hippocrepis	3	Portugal – Portalegre	DB18	39.432 / -7.578	-	-	-	OL547524	OL547560	This study
H. hippocrepis	4	Spain – Toledo	DB25	39.557 / -4.653	-	-	-	OL547525	OL547561	This study
H. hippocrepis	5	Spain – Badajoz	E512.15	38.381 / -6.809	DQ451957	-	-	-	-	Carranza et al. 2006
H. hippocrepis	6	Spain – Huelva	E2509.7	37.266 / -7.018	DQ451960	-	DQ451988	-	-	Carranza et al. 2006
H. hippocrepis	7	Spain – Huelva	E512.14	37.335 / -6.537	DQ451958	-	-	-	-	Carranza et al. 2006
H. hippocrepis	8	Spain – Sevilha	E2509.5	37.614 / -6.171	DQ451962	-	DQ451991	-	-	Carranza et al. 2006
H. hippocrepis	9	Spain – Sevilha	E1209.14	37.389 / -5.979	DQ451967	-	-	-	-	Carranza et al. 2006
H. hippocrepis	10	Spain – Cádiz	DB939	36.218 / -5.859	-	OL547489	-	OL547526	OL547562	This study
H. hippocrepis	10	Spain – Cádiz	E2509.6	36.218 / -5.859	DQ451961	-	DQ451990	-	-	Carranza et al. 2006
H. hippocrepis	11	Spain – Malaga	E512.16	36.866 / -5.263	DQ451956	-	-	-	-	Carranza et al. 2006
H. hippocrepis	12	Spain – Malaga	E512.17	36.366 / -5.227	DQ451955	-	-	-	-	Carranza et al. 2006
H. hippocrepis	13	Spain – Malaga	E512.8	37.183 / -4.334	DQ451982	-	-	-	-	Carranza et al. 2006
H. hippocrepis	14	Spain – Cordova	DB1210	37.608 / -4.329	-	-	-	OL547527	OL547563	This study
H. hippocrepis	15	Spain – Granada	E512.13	36.7804/ -3.730	DQ451980	-	-	-	-	Carranza et al. 2006
H. hippocrepis	16	Spain – Granada	E512.18	36.943 / -3.382	DQ451981	-	-	-	-	Carranza et al. 2006
H. hippocrepis	17	Spain – Jaen	E1209.11	37.8218/ -3.412	DQ451968	-	-	-	-	Carranza et al. 2006
H. hippocrepis	18	Spain – Jaen	DB1790	38.285 / -2.648	-	-	-	OL547528	OL547564	This study
H. hippocrepis	19	Spain – Jaen	E512.9	37.975 / -2.759	DQ451983	-	-	-	-	Carranza et al. 2006
H. hippocrepis	20	Spain – Almeria	E512.11	37.365 / -1.668	DQ451979	-	-	-	-	Carranza et al. 2006
H. hippocrepis	21	Spain – Alicante	DB3828	38.143 / -0.780	OL547485	-	-	OL547529	OL547565	This study
H. hippocrepis	22	Spain – Murcia	DB1299	38.264 / -1.189	-	-	-	OL547530	-	This study
H. hippocrepis	23	Spain – Lerida	E1209.12	41.909 / 0.864	DQ451976	-	-	-	-	Carranza et al. 2006
H. hippocrepis	24	Spain – Lerida	E1209.13	42.166 / 0.895	DQ451977	-	DQ451999	-	-	Carranza et al. 2006
H. hippocrepis	25	Spain – Barcelona	E1209.9	41.645 / 2.743	DQ451978	-	DQ452000	-	-	Carranza et al. 2006
H. hippocrepis	26	Morocco – Tanger-Tétouan-Al Hoceima	E2509.3	35.298 / -5.613	DQ451964	-	DQ452002	-	-	Carranza et al. 2006
H. hippocrepis	27	Morocco – Tanger-Tétouan-Al Hoceima	E512.6	35.606 / -5.633	DQ451953	-	-	-	-	Carranza et al. 2006

Species	Locality Code	Country – Region/District	Sample Code	Latitude / Longitude	cyt-b (short)	cyt-b (long)	12S	MC1R	BDNF	Reference
H. hippocrepis	28	Morocco – Tanger-Tétouan-Al Hoceima	E512.7	35.588 / -5.362	DQ451974	-	DQ451997	-	-	Carranza et al. 2006
H. hippocrepis	29	Morocco – Tanger-Tétouan-Al Hoceima	E2509.1	35.415 / -5.406	DQ451965	-	DQ451993	-	-	Carranza et al. 2006
H. hippocrepis	29	Morocco – Tanger-Tétouan-Al Hoceima	E2509.2	35.453 / -5.392	AY643392	-	AY643308	-	-	Carranza et al. 2006
H. hippocrepis	30	Morocco – Tanger-Tétouan-Al Hoceima	E512.5	35.163 / -5.265	DQ451952	-	-	-	-	Carranza et al. 2006
H. hippocrepis	31	Morocco – Tanger-Tétouan-Al Hoceima	E512.4	35.320 / -6.070	DQ451954	-	DQ451989	-	-	Carranza et al. 2006
H. hippocrepis	31	Morocco – Tanger-Tétouan-Al Hoceima	E512.1	35.320 / -6.070	DQ451959	-	-	-	-	Carranza et al. 2006
H. hippocrepis	32	Morocco – Rabat-Salé-Kénitra	E1209.8	34.861 / -5.913	DQ451966	-	-	-	-	Carranza et al. 2006
H. hippocrepis	32	Morocco – Rabat-Salé-Kénitra	DB2338	34.771 / -6.087			OL547510			This study
H. hippocrepis	33	Morocco – Tanger-Tétouan-Al Hoceima	DB2330	34.795 / -5.559	-	-	OL547511	-	OL547566	This study
H. hippocrepis	34	Morocco – Tanger-Tétouan-Al Hoceima	E512.3	35.083 / -4.618	DQ451969	-	-	-	-	Carranza et al. 2006
H. hippocrepis	35	Morocco – Tanger-Tétouan-Al Hoceima	DB3933	35.040 / -3.821	-	-	OL547512	OL547531	OL547567	This study
H. hippocrepis	36	Morocco -L'Oriental	DB3934	35.088 / -2.477	-	-	-	-	OL547568	This study
H. hippocrepis	36	Morocco -L'Oriental	DB3935	35.080 / -2.491	-	-	-	OL547532	OL547569	This study
H. hippocrepis	36	Morocco -L'Oriental	E2509.4	35.115 / -2.431	DQ451963	-	DQ451992	-	-	Carranza et al. 2006
H. hippocrepis	37	Algeria – Tlemcen	E1110.7	34.678 / -1.367	DQ451973	-	DQ451996	-	-	Carranza et al. 2006
H. hippocrepis	38	Morocco – Fès-Meknès	DB3932	34.047 / -5.325	-	-	-	-	OL547570	This study
H. hippocrepis	39	Morocco – Fès-Meknès	E5113.2	34.023 / -4.993	DQ451970	-	DQ451998	-	-	Carranza et al. 2006
H. hippocrepis	40	Morocco – Fès-Meknès	DB3873	34.130/ -4.029	-	-	-	-	OL547571	This study
H. hippocrepis	40	Morocco – Fès-Meknès	DB3874	34.104/ -4.072	-	OL547490	-	-	OL547572	This study
H. hippocrepis	40	Morocco – Fès-Meknès	DB3886	34.128/ -4.033	-	OL547491	-	OL547533	OL547573	This study
H. hippocrepis	41	Morocco – Fès-Meknès	E5113.1	33.434 / -5.231	DQ451984	-	DQ452001	-	-	Carranza et al. 2006
H. hippocrepis	42	Morocco – Béni Mellal-Khénifra	DB1203	32.609 / -6.282	-	-	-	OL547534	OL547574	This study
H. hippocrepis	43	Morocco – Béni Mellal-Khénifra	DB79	32.489 / -5.930	-	OL547492	OL547513	OL547535	OL547575	This study
H. hippocrepis	44	Morocco – Béni Mellal-Khénifra	DB135	32.460 / -5.785	-	OL547493	-	OL547536	OL547576	This study

Species	Locality Code	Country – Region/District	Sample Code	Latitude / Longitude	cyt-b (short)	cyt-b (long)	12S	MC1R	BDNF	Reference
H. hippocrepis	45	Morocco – Marrakech-Safi	DB11041	31.889 / -7.942	-	-	-	OL547537	-	This study
H. hippocrepis	46	Morocco – Marrakech-Safi	E5113.3	31.629 / -7.981	DQ451975	-	DQ452003	-	-	Carranza et al. 2006
H. hippocrepis	47	Morocco – Drâa-Tafilalet	DB3936	30.916 / -7.236	-	-	-	OL547538		This study
H. hippocrepis	48	Algeria – Alger	E1110.5	36.655 / 2.825	DQ451971	-	DQ451994	-	-	Carranza et al. 2006
H. hippocrepis	49	Algeria – Tizi Ouzou	ZCDB932	36.545 / 4.039	-	OL547494	-	OL547539	OL547577	This study
H. hippocrepis	50	Algeria – M'sila	E1110.6	35.314 / 4.218	DQ451972	-	DQ451995	-	-	Carranza et al. 2006
H. hippocrepis	51	Algeria – Batna	ZCDB922	35.404/ 6.394	-	OL547495	OL547514	OL547540	OL547578	This study
H. hippocrepis	52	Tunisia – Jendouba	E2509.8	36.767 / 8.588	DQ451986	-	DQ452006	-	-	Carranza et al. 2006
H. hippocrepis	53	Tunisia – Jendouba	DB7	36.464 / 8.740	-	OL547496	-	OL547541	OL547579	This study
H. hippocrepis	54	Tunisia – Kef	E2509.10	36.295 / 8.767	DQ451985	-	DQ452004	-	-	Carranza et al. 2006
H. hippocrepis	54	Tunisia – Kef	E2509.9	36.295 / 8.767	DQ451987	-	DQ452005	-	-	Carranza et al. 2006
H. hippocrepis	55	Tunisia – Béja	DB1	36.545 / 9.413	OL547486	-	OL547515	OL547542	OL547580	This study
M. monspessulanus	1	Portugal – Porto	DB13828	41.339 / -8.465	OL547487	-	-	-	-	This study
M. monspessulanus	2	Portugal – Braga	DB13175	41.351 / -8.296	-	-	-	-	OL547581	This study
M. monspessulanus	3	Portugal – Viseu	DB11293	40.978 / -8.014	-	-	-	OL547543	OL547582	This study
M. monspessulanus	4	Portugal – Vila Real	DB3864	41.492 / -7.621	-	-	-	OL547544	OL547583	This study
M. monspessulanus	5	Portugal – Castelo Branco	DB2322	39.814 / -6.998	-	-	-	OL547545	OL547584	This study
M. monspessulanus	6	Portugal – Beja	DB450	37.589 / -7.957	-	OL547497	-	OL547546	OL547585	This study
M. monspessulanus	7	Spain – Huelva	E5121.54	37.247 / -6.940	DQ451907	-	DQ451933	-	-	Carranza et al. 2006
M. monspessulanus	8	Spain – Huelva	E5121.56	38.138 / -6.871	DQ451905	-	-	-	-	Carranza et al. 2006
M. monspessulanus	9	Spain – Huelva	E2509.22	37.162 / -6.666	DQ451894	-	-	-	-	Carranza et al. 2006
M. monspessulanus	10	Spain – Sevilha	E5121.44	37.387 / -6.201	DQ451914	-	-	-	-	Carranza et al. 2006
M. monspessulanus	11	Spain – Sevilha	E14053.12	37.389 / -5.984	DQ451921	-	DQ451938	-	-	Carranza et al. 2006
M. monspessulanus	12	Spain – Sevilha	E1209.5	37.127 / -6.119	DQ451892	-	DQ451931	-	-	Carranza et al. 2006
M. monspessulanus	13	Spain – Cadiz	E1110.18	36.685 / -6.126	DQ451890	-	-	-	-	Carranza et al. 2006
M. monspessulanus	13	Spain – Cadiz	E2509.20	36.621 / -6.057	DQ451897	-	-	-	-	Carranza et al. 2006
M. monspessulanus	14	Spain – Cadiz	E14053.10	36.606 / -5.802	DQ451901	-	DQ451936	-	-	Carranza et al. 2006
M. monspessulanus	15	Spain – Cadiz	E1110.19	36.177 / -5.834	DQ451891	-	-	-	-	Carranza et al. 2006
M. monspessulanus	16	Spain – Cadiz	E1209.7	36.189 / -5.922	DQ451889	-	-	-	-	Carranza et al. 2006
M. monspessulanus	16	Spain – Cadiz	E1110.17	36.257 / -5.966	DQ451895	-	-	-	-	Carranza et al. 2006
M. monspessulanus	17	Spain – Malaga	E5121.49	36.735 / -5.251	DQ451911	-	-	-	-	Carranza et al. 2006
M. monspessulanus	18	Spain – Granada	E5121.46	37.183 / -4.334	DQ451916	-	-	-	-	Carranza et al. 2006
M. monspessulanus	19	Spain – Granada	E5121.43	37.132 / -3.822	DQ451912	-	-	-	-	Carranza et al. 2006
M. monspessulanus	20	Spain – Granada	E5121.47	36.980 / -3.728	DQ451917	-	-	-	-	Carranza et al. 2006
M. monspessulanus	21	Spain – Granada	E5121.55	37.019 / -3.514	DQ451904	-	-	-	-	Carranza et al. 2006

Species	Locality Code	Country – Region/District	Sample Code	Latitude / Longitude	cyt-b (short)	cyt-b (long)	12S	MC1R	BDNF	Reference
M. monspessulanus	22	Spain – Granada	E1209.4	37.222 / -3.515	DQ451924	-	DQ451941	-	-	Carranza et al. 2006
M. monspessulanus	22	Spain – Granada	E1209.3	37.222 / -3.514	DQ451913	-	-	-	-	Carranza et al. 2006
M. monspessulanus	23	Spain – Jaen	E5121.48	37.801 / -3.495	DQ451910	-	-	-	-	Carranza et al. 2006
M. monspessulanus	24	Spain – Jaen	E5121.53	37.764 / -3.077	DQ451906	-	-	-	-	Carranza et al. 2006
M. monspessulanus	25	Spain – Almeria	E5121.50	37.033 / -2.621	DQ451923	-	DQ451940	-	-	Carranza et al. 2006
M. monspessulanus	26	Spain – Almeria	E5121.52	37.052 / -2.387	DQ451909	-	-	-	-	Carranza et al. 2006
M. monspessulanus	27	Spain – Almeria	E5121.57	36.845 / -2.043	DQ451918	-	-	-	-	Carranza et al. 2006
M. monspessulanus	28	Spain – Almeria	E5121.45	37.359 / -1.657	DQ451915	-	DQ451935	-	-	Carranza et al. 2006
M. monspessulanus	28	Spain – Almeria	E5121.51	37.365 / -1.668	DQ451908	-	-	-	-	Carranza et al. 2006
M. monspessulanus	29	Spain – Murcia	DB3855	37.627 / -1.157	-	OL547498	OL547516	-	-	This study
M. monspessulanus	30	Spain – Murcia	DB3840	37.727 / -0.707	-	-	-	OL547547	-	This study
M. monspessulanus	31	Spain – Alicante	DB3826	38.244 / -0.524	-	OL547499	OL547517	OL547548	OL547586	This study
M. monspessulanus	32	Spain – Alicante	DB3821	38.413 / -0.445	-	-	-	OL547549	OL547587	This study
M. monspessulanus	33	Spain – Albacete	DB3856	38.168 / -2.271	-	-	OL547518	OL547550	OL547588	This study
M. monspessulanus	34	Spain – Ciudad Real	DB28	39.050 / -2.959	-	OL547500	-	OL547551	OL547589	This study
M. monspessulanus	35	Spain – Barcelona	E14053.9	41.450 / 2.247	DQ451900	-	DQ451939	-	-	Carranza et al. 2006
M. monspessulanus	36	Spain – Barcelona	E14053.11	41.689 / 2.489	DQ451898	-	DQ451937	-	-	Carranza et al. 2006
M. monspessulanus	37	Morocco – Tanger-Tétouan-Al Hoceima	E1209.6	35.759 / -5.833	DQ451888	-	-	-	-	Carranza et al. 2006
M. monspessulanus	38	Spain – Ceuta	E2509.21	35.849 / -5.382	DQ451920	-	DQ451928	-	-	Carranza et al. 2006
M. monspessulanus	38	Spain – Ceuta	E2509.19	35.889 / -5.321	DQ451896	-	-	-	-	Carranza et al. 2006
M. monspessulanus	39	Morocco – Rabat-Salé-Kénitra	E2509.15	34.679 / -6.011	AY643396	-	-	-	-	Carranza et al. 2006
M. monspessulanus	40	Morocco – Rabat-Salé-Kénitra	E5113.13	34.388 / -6.513	DQ451899	-	DQ451929	-	-	Carranza et al. 2006
M. monspessulanus	41	Morocco – Tanger-Tétouan-Al Hoceima	DB2325	34.768 / -5.516	-	OL547501	-	OL547552	-	This study
M. monspessulanus	42	Morocco -L'Oriental	E2509.16	35.1475 / -2.96	DQ451922	-	DQ451934	-	-	Carranza et al. 2006
M. monspessulanus	43	Morocco -L'Oriental	E2509.18	35.055 / -2.666	DQ451903	-	DQ451927	-	-	Carranza et al. 2006
M. monspessulanus	44	Morocco -L'Oriental	E14053.2	35.043 / -2.317	DQ451902	-	DQ451930	-	-	Carranza et al. 2006
M. monspessulanus	45	Morocco – Fès-Meknès	DB3881	33.439 / -5.166	-	OL547502	-	OL547553	OL547590	This study
M. monspessulanus	46	Morocco – Tadla-Azilal – Béni Mellal-Khénifra	DB1201	32.442 / -5.988	-	OL547503	OL547519	OL547554	OL547591	This study
M. monspessulanus	47	Morocco – Tadla-Azilal – Béni Mellal-Khénifra	DB3351	32.271 / -6.501	-	OL547504	OL547520	OL547555	OL547592	This study
M. monspessulanus	48	Morocco – Marrakech-Safi	DB11973	31.539 / -9.504	-	OL547505	OL547521	-	OL547593	This study

Species	Locality Code	Country – Region/District	Sample Code	Latitude / Longitude	cyt-b (short)	cyt-b (long)	12S	MC1R	BDNF	Reference
M. monspessulanus	49	Morocco – Marrakech-Safi	E2509.17	31.391 / -9.447	DQ451925	-	DQ451943	-	-	Carranza et al. 2006
M. monspessulanus	50	Morocco – Drâa-Tafilalet	DB11000	30.799 / -7.527	-	OL547506	-	OL547556	OL547594	This study
M. monspessulanus	50	Morocco – Drâa-Tafilalet	DB9253	30.808 / -7.583	-	OL547507	OL547522	-	OL547595	This study
M. monspessulanus	51	Algeria – Naâma	E9124.1	33.938 / -1.113	DQ451893	-	DQ451942	-	-	Carranza et al. 2006
M. monspessulanus	52	Algeria – Tiaret	ZCDB877	35.383 / 1.350	-	OL547508	OL547523	OL547557	-	This study
M. monspessulanus	53	Algeria – Médéa	E9124.2	35.816 / 2.794	DQ451919	-	DQ451932	-	-	Carranza et al. 2006

Table S2 - Summary statistics and substitution models used for the Extended Bayesian Skyline Plot analyses.

Species	Partition	Nº Individuals	Length	Variable sites	Parsimony Informative sites	Substitution Model
H. hippocrepis	cyt-b	51	279	15	9	HKY
	12S	51	358	5	3	HKY+I
	MC1R	38	574	1	0	HKY
	BDNF	46	561	2	2	HKY
M. monspessulanus	cyt-b	53	282	13	5	HKY
	12S	53	377	8	3	HKY
	MC1R	30	612	2	0	HKY
	BDNF	30	534	4	2	HKY

Table S3: Occurrence data for *H. hippocrepis* used as input for Maxent modelling analysis.

Species	X	Y
H. hippocrepis	-7.2000	39.2400
H. hippocrepis	-7.0200	37.4400
H. hippocrepis	-7.0100	37.8000
H. hippocrepis	-7.0100	37.9800
H. hippocrepis	-6.8400	39.6800
H. hippocrepis	-6.8000	40.9500
H. hippocrepis	-6.7100	40.2200
H. hippocrepis	-6.6900	37.1600
H. hippocrepis	-6.6800	37.4300
H. hippocrepis	-6.6800	41.0300
H. hippocrepis	-6.6700	37.7000
H. hippocrepis	-6.4400	37.8700
H. hippocrepis	-6.4300	41.3000
H. hippocrepis	-6.3400	37.5100
H. hippocrepis	-6.3300	37.7800
H. hippocrepis	-6.2800	39.1300
H. hippocrepis	-6.2100	37.9600
H. hippocrepis	-6.1800	38.6800
H. hippocrepis	-6.1200	37.3300
H. hippocrepis	-6.0700	38.5900
H. hippocrepis	-6.0600	41.5600
H. hippocrepis	-6.0300	36.8700
H. hippocrepis	-6.0200	37.1400
H. hippocrepis	-5.8400	36.3400
H. hippocrepis	-5.7400	36.6100
H. hippocrepis	-5.7200	36.0700
H. hippocrepis	-5.6200	36.2500
H. hippocrepis	-5.5500	37.5100
H. hippocrepis	-5.5400	37.2400
H. hippocrepis	-5.5300	36.9700
H. hippocrepis	-5.5300	40.4000
H. hippocrepis	-5.5100	36.3400
H. hippocrepis	-5.5000	39.5000
H. hippocrepis	-5.4400	37.6100
H. hippocrepis	-5.4200	37.0700
H. hippocrepis	-5.4100	40.1300
H. hippocrepis	-5.3100	37.2500
H. hippocrepis	-5.2300	38.3300
H. hippocrepis	-5.2100	37.6100
H. hippocrepis	-5.1700	39.9600
H. hippocrepis	-5.1500	39.5900
H. hippocrepis	-5.1400	39.2300
H. hippocrepis	-5.1100	38.1500
H. hippocrepis	-5.0900	37.2500
H. hippocrepis	-5.0800	36.8900
H. hippocrepis	-5.0400	39.6900
H. hippocrepis	-4.8700	37.8000
H. hippocrepis	-4.8400	36.5400
H. hippocrepis	-4.8000	39.4200
H. hippocrepis	-4.7700	38.3400
H. hippocrepis	-4.6800	39.3300
H. hippocrepis	-4.6500	37.7100

Species	X	Y
H. hippocrepis	-4.6300	36.8100
H. hippocrepis	-4.5500	38.4300
H. hippocrepis	-4.5300	37.5300
H. hippocrepis	-4.5100	36.7200
H. hippocrepis	-4.4100	37.5300
H. hippocrepis	-4.3400	39.5200
H. hippocrepis	-4.3300	39.3400
H. hippocrepis	-4.2900	36.9900
H. hippocrepis	-4.2400	40.3300
H. hippocrepis	-4.1900	37.7200
H. hippocrepis	-3.9700	37.9900
H. hippocrepis	-3.8400	36.9100
H. hippocrepis	-3.7700	40.4200
H. hippocrepis	-3.7400	37.7200
H. hippocrepis	-3.7300	37.0900
H. hippocrepis	-3.7300	37.4500
H. hippocrepis	-3.6400	39.3400
H. hippocrepis	-3.6318	38.6212
H. hippocrepis	-3.6300	38.3500
H. hippocrepis	-3.6200	37.1800
H. hippocrepis	-3.6200	37.4500
H. hippocrepis	-3.6200	37.5400
H. hippocrepis	-3.4000	37.4500
H. hippocrepis	-3.4000	38.4400
H. hippocrepis	-3.3900	36.9100
H. hippocrepis	-3.2800	37.9000
H. hippocrepis	-3.0600	38.1700
H. hippocrepis	-3.0600	38.7100
H. hippocrepis	-9.4025	38.7123
H. hippocrepis	-9.2894	39.1632
H. hippocrepis	-9.2886	38.9830
H. hippocrepis	-9.2875	38.7126
H. hippocrepis	-8.9420	39.3438
H. hippocrepis	-8.9418	39.5240
H. hippocrepis	-8.8306	37.3610
H. hippocrepis	-8.8255	39.5238
H. hippocrepis	-8.7187	37.0904
H. hippocrepis	-8.7153	37.9917
H. hippocrepis	-8.5923	39.6134
H. hippocrepis	-8.4931	37.1797
H. hippocrepis	-8.3601	39.5222
H. hippocrepis	-8.2678	37.1786
H. hippocrepis	-8.2625	37.7193
H. hippocrepis	-8.2571	38.2600
H. hippocrepis	-8.2307	40.6926
H. hippocrepis	-8.1531	37.3581
H. hippocrepis	-8.1480	37.8087
H. hippocrepis	-8.1459	37.9889
H. hippocrepis	-8.1195	40.1513
H. hippocrepis	-8.0425	37.1770
H. hippocrepis	-8.0391	37.4473
H. hippocrepis	-8.0200	38.8891
H. hippocrepis	-8.0073	39.7900
H. hippocrepis	-7.9312	37.0859
H. hippocrepis	-7.9209	37.8068

Species	X	Y
H. hippocrepis	-7.9169	38.0772
H. hippocrepis	-7.8934	39.6088
H. hippocrepis	-7.8087	37.7156
H. hippocrepis	-7.7999	38.2563
H. hippocrepis	-7.7640	40.3282
H. hippocrepis	-7.6873	38.1649
H. hippocrepis	-7.6708	39.0659
H. hippocrepis	-7.5767	37.9834
H. hippocrepis	-7.5679	38.4339
H. hippocrepis	-7.5248	40.5056
H. hippocrepis	-7.4629	37.9820
H. hippocrepis	-7.4553	38.3423
H. hippocrepis	-7.4436	38.8828
H. hippocrepis	-7.4397	39.0630
H. hippocrepis	-7.4235	39.7836
H. hippocrepis	-7.3916	41.1344
H. hippocrepis	-7.3347	38.6110
H. hippocrepis	-7.3090	39.6919
H. hippocrepis	-7.3001	40.0521
H. hippocrepis	-7.2956	40.2322
H. hippocrepis	-7.2842	40.6824
H. hippocrepis	-7.1901	39.7802
H. hippocrepis	-7.0658	40.0484
H. hippocrepis	-6.9540	39.8663
H. hippocrepis	-6.9350	40.4966
H. hippocrepis	-2.8300	37.1800
H. hippocrepis	-2.8300	38.0800
H. hippocrepis	-2.4734	36.8427
H. hippocrepis	-2.3800	37.0900
H. hippocrepis	-2.3800	37.8100
H. hippocrepis	-2.3700	38.2600
H. hippocrepis	-2.2700	36.8200
H. hippocrepis	-2.1600	36.9100
H. hippocrepis	-2.1500	37.8100
H. hippocrepis	-2.1400	38.5300
H. hippocrepis	-2.0400	37.3600
H. hippocrepis	-1.9300	37.0900
H. hippocrepis	-1.6900	38.2600
H. hippocrepis	-1.5800	37.8900
H. hippocrepis	-1.3500	37.9800
H. hippocrepis	-1.3400	38.1600
H. hippocrepis	-1.2000	39.2400
H. hippocrepis	-1.1900	39.9600
H. hippocrepis	-1.1000	38.7000
H. hippocrepis	-1.0800	39.4200
H. hippocrepis	-0.8400	39.6800
H. hippocrepis	-0.7700	38.1500
H. hippocrepis	-0.6600	38.2400
H. hippocrepis	-0.6000	39.9500
H. hippocrepis	-0.5400	38.2400
H. hippocrepis	-0.5000	39.5000
H. hippocrepis	-0.4800	39.9500
H. hippocrepis	-0.4100	38.7800
H. hippocrepis	-0.2900	38.8600
H. hippocrepis	-0.0274	40.3862

Species	X	Y
H. hippocrepis	-0.0100	40.0300
H. hippocrepis	0.0900	40.8400
H. hippocrepis	0.2000	41.1100
H. hippocrepis	0.2100	40.8400
H. hippocrepis	0.2300	40.3000
H. hippocrepis	0.4500	40.7600
H. hippocrepis	0.6800	41.1200
H. hippocrepis	0.6836	40.9422
H. hippocrepis	0.8900	41.8500
H. hippocrepis	1.1500	41.2200
H. hippocrepis	1.5100	41.3200
H. hippocrepis	1.6300	41.2300
H. hippocrepis	-10.8710	28.5059
H. hippocrepis	-10.6174	28.6339
H. hippocrepis	-10.2442	29.0557
H. hippocrepis	-10.1759	29.3701
H. hippocrepis	-10.0519	28.9967
H. hippocrepis	-9.7594	31.5085
H. hippocrepis	-9.7383	29.1100
H. hippocrepis	-9.7280	29.7250
H. hippocrepis	-9.6177	31.3312
H. hippocrepis	-9.5747	30.0409
H. hippocrepis	-9.5296	30.3039
H. hippocrepis	-9.3957	29.8225
H. hippocrepis	-9.2885	31.8009
H. hippocrepis	-9.1763	32.5055
H. hippocrepis	-9.0700	31.5385
H. hippocrepis	-9.0483	30.7801
H. hippocrepis	-9.0419	29.6664
H. hippocrepis	-9.0132	30.4095
H. hippocrepis	-8.7978	31.1573
H. hippocrepis	-8.7868	30.8305
H. hippocrepis	-8.7411	31.7773
H. hippocrepis	-8.6406	32.9183
H. hippocrepis	-8.4952	31.3283
H. hippocrepis	-8.3653	31.8863
H. hippocrepis	-8.3300	31.6524
H. hippocrepis	-8.3070	30.7547
H. hippocrepis	-8.1648	32.2249
H. hippocrepis	-8.0907	31.3531
H. hippocrepis	-8.0251	32.8384
H. hippocrepis	-7.9562	31.5789
H. hippocrepis	-7.9423	31.8894
H. hippocrepis	-7.9068	31.2977
H. hippocrepis	-7.8706	32.6607
H. hippocrepis	-7.8600	32.1664
H. hippocrepis	-7.6469	31.5419
H. hippocrepis	-7.6364	31.8438
H. hippocrepis	-7.6242	32.6281
H. hippocrepis	-7.5770	32.8387
H. hippocrepis	-7.5531	33.5926
H. hippocrepis	-7.3622	33.6349
H. hippocrepis	-7.3212	31.4041
H. hippocrepis	-7.2364	30.9165
H. hippocrepis	-7.1740	33.0600

Species	X	Y
H. hippocrepis	-7.1049	33.3437
H. hippocrepis	-7.0830	33.5834
H. hippocrepis	-7.0686	33.7842
H. hippocrepis	-7.0356	31.7863
H. hippocrepis	-6.7621	33.9995
H. hippocrepis	-6.6324	34.2064
H. hippocrepis	-6.6284	32.2630
H. hippocrepis	-6.5871	33.5496
H. hippocrepis	-6.5758	31.9274
H. hippocrepis	-6.5369	31.7777
H. hippocrepis	-6.3575	34.1896
H. hippocrepis	-6.3147	33.6661
H. hippocrepis	-6.2947	34.8728
H. hippocrepis	-6.2821	32.6100
H. hippocrepis	-6.0866	34.7707
H. hippocrepis	-6.0702	35.3206
H. hippocrepis	-6.0212	33.3364
H. hippocrepis	-5.9308	32.4894
H. hippocrepis	-5.8128	35.7847
H. hippocrepis	-5.7100	35.6300
H. hippocrepis	-5.6131	35.2984
H. hippocrepis	-5.5915	32.1060
H. hippocrepis	-5.5590	34.7960
H. hippocrepis	-5.5509	35.8475
H. hippocrepis	-5.5192	32.6072
H. hippocrepis	-5.4149	35.0701
H. hippocrepis	-5.4069	35.4150
H. hippocrepis	-5.3800	35.9000
H. hippocrepis	-5.3529	33.0847
H. hippocrepis	-5.3253	34.0473
H. hippocrepis	-5.3169	33.5427
H. hippocrepis	-5.2942	35.6805
H. hippocrepis	-5.2784	34.5909
H. hippocrepis	-5.2697	35.1714
H. hippocrepis	-5.2468	33.8366
H. hippocrepis	-5.1607	34.3737
H. hippocrepis	-5.1468	32.2621
H. hippocrepis	-5.1245	34.6885
H. hippocrepis	-5.0960	33.1378
H. hippocrepis	-5.0933	32.9380
H. hippocrepis	-5.0827	35.4111
H. hippocrepis	-4.9938	34.0230
H. hippocrepis	-4.9484	35.1769
H. hippocrepis	-4.9183	34.6695
H. hippocrepis	-4.8626	33.4944
H. hippocrepis	-4.8344	35.0149
H. hippocrepis	-4.8157	33.7969
H. hippocrepis	-4.6551	34.9658
H. hippocrepis	-4.6478	35.1732
H. hippocrepis	-4.6014	34.5707
H. hippocrepis	-4.5361	32.5907
H. hippocrepis	-4.4415	35.1381
H. hippocrepis	-4.1521	35.0658
H. hippocrepis	-4.0292	34.1305
H. hippocrepis	-3.9345	34.7045

Species	X	Y
H. hippocrepis	-3.8216	35.0409
H. hippocrepis	-3.7023	34.8507
H. hippocrepis	-3.6377	35.1154
H. hippocrepis	-3.6338	34.6052
H. hippocrepis	-3.5871	33.5401
H. hippocrepis	-3.3989	34.9374
H. hippocrepis	-3.3763	34.4610
H. hippocrepis	-3.1400	33.8473
H. hippocrepis	-2.9983	35.3249
H. hippocrepis	-2.9611	34.0960
H. hippocrepis	-2.8903	35.0437
H. hippocrepis	-2.8874	34.3848
H. hippocrepis	-2.7777	32.6878
H. hippocrepis	-2.5842	34.9303
H. hippocrepis	-2.4775	35.0888
H. hippocrepis	-2.4350	34.8082
H. hippocrepis	-2.3092	34.9015
H. hippocrepis	-2.2053	34.0535
H. hippocrepis	-2.0676	34.3614
H. hippocrepis	-1.8562	35.0962
H. hippocrepis	-1.3670	34.6782
H. hippocrepis	-1.3396	34.1922
H. hippocrepis	-1.3139	34.8825
H. hippocrepis	-0.6309	35.6969
H. hippocrepis	-0.6299	35.2022
H. hippocrepis	-0.6298	35.2020
H. hippocrepis	0.1549	35.4804
H. hippocrepis	0.5400	32.9032
H. hippocrepis	0.8695	33.7076
H. hippocrepis	2.8251	36.6557
H. hippocrepis	2.9976	36.4206
H. hippocrepis	3.0333	35.1500
H. hippocrepis	3.0421	36.7529
H. hippocrepis	3.0777	35.4172
H. hippocrepis	3.6666	36.8291
H. hippocrepis	3.9555	36.5410
H. hippocrepis	4.1099	36.6775
H. hippocrepis	4.2181	35.3149
H. hippocrepis	4.2703	36.8163
H. hippocrepis	4.2714	36.4909
H. hippocrepis	4.4248	36.7534
H. hippocrepis	6.0891	35.1281
H. hippocrepis	6.3948	35.4049
H. hippocrepis	6.4284	35.1862
H. hippocrepis	7.1465	35.4309
H. hippocrepis	7.7525	36.9265
H. hippocrepis	7.9593	35.1291
H. hippocrepis	8.5977	36.7647
H. hippocrepis	8.7404	36.4646
H. hippocrepis	9.4137	36.5459
H. hippocrepis	9.4138	36.5459
H. hippocrepis	9.7870	37.0381
H. hippocrepis	10.3000	36.9000

Table S4 - Occurrence data for *M. monspessulanus* used as input for Maxent modelling analysis

Species	X	Y
M. monspessulanus	-12.8873	27.6071
M. monspessulanus	-12.8208	27.8866
M. monspessulanus	-12.3515	28.0184
M. monspessulanus	-12.0723	28.0173
M. monspessulanus	-11.6551	28.1709
M. monspessulanus	-11.2498	28.5365
M. monspessulanus	-10.8827	28.7709
M. monspessulanus	-10.6713	28.8118
M. monspessulanus	-10.3931	29.0514
M. monspessulanus	-10.1357	29.3246
M. monspessulanus	-10.1034	29.1494
M. monspessulanus	-10.0615	28.9797
M. monspessulanus	-9.9059	29.6659
M. monspessulanus	-9.8015	30.9917
M. monspessulanus	-9.7717	31.5142
M. monspessulanus	-9.7344	29.1863
M. monspessulanus	-9.7333	29.7076
M. monspessulanus	-9.6411	30.0661
M. monspessulanus	-9.6000	30.4000
M. monspessulanus	-9.5046	31.5398
M. monspessulanus	-9.4379	29.8478
M. monspessulanus	-9.2464	30.7282
M. monspessulanus	-9.1819	32.5253
M. monspessulanus	-9.1743	29.9490
M. monspessulanus	-9.0897	32.1026
M. monspessulanus	-9.0509	30.4447
M. monspessulanus	-8.8701	32.8432
M. monspessulanus	-8.8425	31.1485
M. monspessulanus	-8.8162	31.8020
M. monspessulanus	-8.8078	29.6760
M. monspessulanus	-8.6982	30.5665
M. monspessulanus	-8.6248	33.0726
M. monspessulanus	-8.4629	32.6156
M. monspessulanus	-8.4366	30.5800
M. monspessulanus	-8.4253	32.8724
M. monspessulanus	-8.4194	31.9187
M. monspessulanus	-8.3898	31.4200
M. monspessulanus	-8.3897	30.7809
M. monspessulanus	-8.3798	31.6677
M. monspessulanus	-8.1851	31.8534
M. monspessulanus	-8.0486	31.6262
M. monspessulanus	-7.9422	31.9033
M. monspessulanus	-7.9222	30.5624
M. monspessulanus	-7.8702	31.1077
M. monspessulanus	-7.8547	31.3553
M. monspessulanus	-7.8167	33.4854
M. monspessulanus	-7.7049	31.5945
M. monspessulanus	-7.6887	31.8528
M. monspessulanus	-7.6876	32.0790
M. monspessulanus	-7.6593	32.6535
M. monspessulanus	-7.6144	33.6051
M. monspessulanus	-7.5837	30.8081
M. monspessulanus	-7.5505	30.4403

Species	X	Y
M. monspessulanus	-7.4439	31.4013
M. monspessulanus	-7.3484	33.0386
M. monspessulanus	-7.3201	31.0658
M. monspessulanus	-7.3008	33.7461
M. monspessulanus	-7.0736	33.3279
M. monspessulanus	-6.9814	31.6928
M. monspessulanus	-6.9734	30.8921
M. monspessulanus	-6.9503	33.8988
M. monspessulanus	-6.9114	33.0744
M. monspessulanus	-6.8129	33.7121
M. monspessulanus	-6.8044	34.0444
M. monspessulanus	-6.7115	32.0304
M. monspessulanus	-6.6686	34.2362
M. monspessulanus	-6.6618	30.6966
M. monspessulanus	-6.5919	32.8571
M. monspessulanus	-6.5535	34.3788
M. monspessulanus	-6.5016	32.2713
M. monspessulanus	-6.4839	31.6389
M. monspessulanus	-6.4698	32.0813
M. monspessulanus	-6.4069	34.6259
M. monspessulanus	-6.3825	34.1316
M. monspessulanus	-6.3503	33.8939
M. monspessulanus	-6.3140	32.5647
M. monspessulanus	-6.3124	34.8398
M. monspessulanus	-6.1102	33.4047
M. monspessulanus	-5.9886	32.4421
M. monspessulanus	-5.9678	35.5868
M. monspessulanus	-5.9310	32.0266
M. monspessulanus	-5.8578	35.4559
M. monspessulanus	-5.6675	32.9394
M. monspessulanus	-5.5825	33.1512
M. monspessulanus	-5.5741	34.9153
M. monspessulanus	-5.5000	34.0170
M. monspessulanus	-5.4237	32.3134
M. monspessulanus	-5.3925	33.6787
M. monspessulanus	-5.3728	33.3030
M. monspessulanus	-5.3725	35.5964
M. monspessulanus	-5.3563	35.3175
M. monspessulanus	-5.2180	35.0719
M. monspessulanus	-5.1915	34.4816
M. monspessulanus	-5.1665	33.4391
M. monspessulanus	-5.1199	32.7823
M. monspessulanus	-5.1000	35.4129
M. monspessulanus	-5.0924	33.6137
M. monspessulanus	-5.0775	34.0507
M. monspessulanus	-5.0352	33.8306
M. monspessulanus	-4.9934	34.6300
M. monspessulanus	-4.9788	32.9042
M. monspessulanus	-4.9623	34.9748
M. monspessulanus	-4.8522	33.7015
M. monspessulanus	-4.8406	33.1250
M. monspessulanus	-4.8028	32.6654
M. monspessulanus	-4.6999	34.9554
M. monspessulanus	-4.6277	32.6200
M. monspessulanus	-4.5186	35.0820

Species	X	Y
M. monspessulanus	-4.2150	34.6490
M. monspessulanus	-4.2057	35.0892
M. monspessulanus	-4.1796	33.3139
M. monspessulanus	-4.0905	34.8917
M. monspessulanus	-4.0902	33.6498
M. monspessulanus	-4.0067	34.2239
M. monspessulanus	-3.9067	34.6465
M. monspessulanus	-3.8229	34.8771
M. monspessulanus	-3.7216	32.6443
M. monspessulanus	-3.7054	34.2144
M. monspessulanus	-3.6210	34.6390
M. monspessulanus	-3.5748	34.9021
M. monspessulanus	-3.4513	34.6266
M. monspessulanus	-3.3833	34.9833
M. monspessulanus	-3.1675	34.3458
M. monspessulanus	-3.1343	34.0471
M. monspessulanus	-3.0274	35.2131
M. monspessulanus	-2.9306	34.6351
M. monspessulanus	-2.9225	33.6772
M. monspessulanus	-2.9014	34.3638
M. monspessulanus	-2.8149	35.1761
M. monspessulanus	-2.7168	34.0984
M. monspessulanus	-2.5762	34.6241
M. monspessulanus	-2.4574	34.0324
M. monspessulanus	-2.3714	35.0523
M. monspessulanus	-2.1880	34.3213
M. monspessulanus	-1.8977	35.0680
M. monspessulanus	-1.4008	34.2069
M. monspessulanus	-1.2662	34.4812
M. monspessulanus	-1.1139	33.9384
M. monspessulanus	0.0667	34.1500
M. monspessulanus	0.4662	35.3990
M. monspessulanus	1.0688	35.3326
M. monspessulanus	1.3500	35.3833
M. monspessulanus	2.7949	35.8164
M. monspessulanus	3.0000	36.8000
M. monspessulanus	3.6666	36.8291
M. monspessulanus	3.8575	36.6183
M. monspessulanus	4.0633	36.7050
M. monspessulanus	4.2704	35.2082
M. monspessulanus	4.2714	36.4909
M. monspessulanus	4.2820	36.8820
M. monspessulanus	5.6333	36.3333
M. monspessulanus	7.7520	35.2704
M. monspessulanus	8.6898	36.7156
M. monspessulanus	8.7333	36.9000
M. monspessulanus	-5.8891	35.8016
M. monspessulanus	-5.3800	35.9000
M. monspessulanus	-9.4041	38.9826
M. monspessulanus	-9.0582	39.6141
M. monspessulanus	-8.9421	39.2536
M. monspessulanus	-8.8310	37.1807
M. monspessulanus	-8.8292	37.9919
M. monspessulanus	-8.7167	37.6312
M. monspessulanus	-8.7084	39.7038

Species	X	Y
M. monspessulanus	-8.7057	40.3345
M. monspessulanus	-8.7045	40.6047
M. monspessulanus	-8.5949	39.1629
M. monspessulanus	-8.5891	40.1539
M. monspessulanus	-8.4913	37.4501
M. monspessulanus	-8.4831	38.6217
M. monspessulanus	-8.4655	41.3390
M. monspessulanus	-8.4563	42.0450
M. monspessulanus	-8.3373	41.8643
M. monspessulanus	-8.2515	38.8007
M. monspessulanus	-8.2297	40.7827
M. monspessulanus	-8.1511	37.5383
M. monspessulanus	-8.1195	40.1513
M. monspessulanus	-8.1136	40.6017
M. monspessulanus	-8.0887	42.4029
M. monspessulanus	-8.0086	39.6999
M. monspessulanus	-7.8727	40.8699
M. monspessulanus	-7.8555	41.8605
M. monspessulanus	-7.7489	41.1388
M. monspessulanus	-7.6464	40.3269
M. monspessulanus	-7.5732	38.1636
M. monspessulanus	-7.5679	38.4339
M. monspessulanus	-7.5552	39.0645
M. monspessulanus	-7.5248	40.5056
M. monspessulanus	-7.5168	40.8658
M. monspessulanus	-7.4740	37.4414
M. monspessulanus	-7.4475	38.7027
M. monspessulanus	-7.4357	39.2432
M. monspessulanus	-7.4297	39.5134
M. monspessulanus	-7.4215	39.8736
M. monspessulanus	-7.3714	41.9447
M. monspessulanus	-7.3450	38.1606
M. monspessulanus	-7.3368	38.5209
M. monspessulanus	-7.2772	40.9526
M. monspessulanus	-7.2725	41.1326
M. monspessulanus	-7.2654	41.4028
M. monspessulanus	-7.2409	42.3030
M. monspessulanus	-7.1283	37.7068
M. monspessulanus	-7.1168	38.1572
M. monspessulanus	-7.1116	42.5711
M. monspessulanus	-7.1075	38.5175
M. monspessulanus	-7.0709	39.8683
M. monspessulanus	-7.0221	37.4347
M. monspessulanus	-6.9405	40.3165
M. monspessulanus	-6.9125	41.2168
M. monspessulanus	-6.7655	42.0245
M. monspessulanus	-6.7365	42.8344
M. monspessulanus	-6.6860	37.3385
M. monspessulanus	-6.5526	37.9666
M. monspessulanus	-6.4920	42.8293
M. monspessulanus	-6.4701	40.3072
M. monspessulanus	-6.4513	37.6040
M. monspessulanus	-6.4262	38.3243
M. monspessulanus	-6.3998	42.1069
M. monspessulanus	-6.2080	38.0490

Species	X	Y
M. monspessulanus	-6.1975	41.2016
M. monspessulanus	-6.1781	41.6514
M. monspessulanus	-6.1256	37.2361
M. monspessulanus	-6.0540	41.9183
M. monspessulanus	-6.0526	36.3331
M. monspessulanus	-6.0442	36.7834
M. monspessulanus	-6.0303	37.5039
M. monspessulanus	-5.9058	38.0462
M. monspessulanus	-5.8508	36.6059
M. monspessulanus	-5.8219	41.6514
M. monspessulanus	-5.8141	41.4715
M. monspessulanus	-5.7540	40.0318
M. monspessulanus	-5.6336	36.7911
M. monspessulanus	-5.6000	42.2000
M. monspessulanus	-5.5997	39.0445
M. monspessulanus	-5.5607	41.1171
M. monspessulanus	-5.5133	39.8571
M. monspessulanus	-5.5070	36.3432
M. monspessulanus	-5.4210	37.1561
M. monspessulanus	-5.4125	36.8859
M. monspessulanus	-5.4059	40.1297
M. monspessulanus	-5.3388	41.5722
M. monspessulanus	-5.3140	37.3385
M. monspessulanus	-5.2950	36.7080
M. monspessulanus	-5.2314	41.9346
M. monspessulanus	-5.1977	40.9445
M. monspessulanus	-5.1600	39.7742
M. monspessulanus	-5.1516	39.5041
M. monspessulanus	-5.0568	40.2265
M. monspessulanus	-5.0250	39.1460
M. monspessulanus	-5.0198	38.9659
M. monspessulanus	-5.0022	38.3354
M. monspessulanus	-4.8267	40.4105
M. monspessulanus	-4.7600	37.8000
M. monspessulanus	-4.7443	41.7629
M. monspessulanus	-4.7204	40.8625
M. monspessulanus	-4.6844	39.4217
M. monspessulanus	-4.6173	41.4945
M. monspessulanus	-4.5603	39.0630
M. monspessulanus	-4.5188	37.0810
M. monspessulanus	-4.4375	38.7042
M. monspessulanus	-4.3430	39.7865
M. monspessulanus	-4.3326	39.2460
M. monspessulanus	-4.3143	38.2550
M. monspessulanus	-4.3047	37.7144
M. monspessulanus	-4.2045	38.5266
M. monspessulanus	-4.1841	37.2650
M. monspessulanus	-4.1785	36.9046
M. monspessulanus	-4.1494	42.1306
M. monspessulanus	-4.1213	40.5096
M. monspessulanus	-4.1052	39.5187
M. monspessulanus	-4.1000	41.7600
M. monspessulanus	-4.0926	38.7079
M. monspessulanus	-4.0871	38.3475
M. monspessulanus	-4.0765	37.6266

Species	X	Y
M. monspessulanus	-3.9563	37.0868
M. monspessulanus	-3.8840	40.4216
M. monspessulanus	-3.8615	38.6197
M. monspessulanus	-3.7366	37.6292
M. monspessulanus	-3.6415	39.7024
M. monspessulanus	-3.6286	38.2607
M. monspessulanus	-3.6188	37.0891
M. monspessulanus	-3.5156	38.4415
M. monspessulanus	-3.5051	36.9093
M. monspessulanus	-3.3991	38.0815
M. monspessulanus	-3.3938	37.0900
M. monspessulanus	-3.2951	40.5146
M. monspessulanus	-3.2943	40.3345
M. monspessulanus	-3.2833	37.6312
M. monspessulanus	-2.9433	37.7216
M. monspessulanus	-2.9418	39.6141
M. monspessulanus	-2.8304	37.4511
M. monspessulanus	-2.7139	38.3522
M. monspessulanus	-2.6962	42.4061
M. monspessulanus	-2.6024	37.8111
M. monspessulanus	-2.4925	37.2699
M. monspessulanus	-2.3752	37.8101
M. monspessulanus	-2.3706	38.3508
M. monspessulanus	-2.3508	40.5132
M. monspessulanus	-2.3428	41.3239
M. monspessulanus	-2.0861	42.5830
M. monspessulanus	-2.0402	37.3572
M. monspessulanus	-2.0237	38.6188
M. monspessulanus	-1.9941	40.6908
M. monspessulanus	-1.9730	42.0417
M. monspessulanus	-1.8029	38.0761
M. monspessulanus	-1.4899	42.0364
M. monspessulanus	-1.3805	41.5846
M. monspessulanus	-1.3531	37.8003
M. monspessulanus	-1.3527	42.6649
M. monspessulanus	-1.2796	40.8625
M. monspessulanus	-1.2654	41.4028
M. monspessulanus	-1.2508	41.9429
M. monspessulanus	-1.2063	39.1498
M. monspessulanus	-1.1948	39.6001
M. monspessulanus	-1.1733	40.4105
M. monspessulanus	-1.1381	41.6710
M. monspessulanus	-1.1122	38.3373
M. monspessulanus	-0.9724	39.2360
M. monspessulanus	-0.9067	41.3968
M. monspessulanus	-0.7322	39.5019
M. monspessulanus	-0.6035	39.8596
M. monspessulanus	-0.5682	40.8497
M. monspessulanus	-0.5345	41.7496
M. monspessulanus	-0.5030	39.4071
M. monspessulanus	-0.4993	42.6494
M. monspessulanus	-0.4735	40.2172
M. monspessulanus	-0.4003	39.0445
M. monspessulanus	-0.3887	42.3768
M. monspessulanus	-0.2917	38.8618

Species	X	Y
M. monspessulanus	-0.2779	39.2218
M. monspessulanus	-0.2350	40.3018
M. monspessulanus	-0.1975	41.2016
M. monspessulanus	-0.1251	40.1190
M. monspessulanus	-0.0652	38.7662
M. monspessulanus	0.0245	40.5667
M. monspessulanus	0.0323	40.2066
M. monspessulanus	0.0903	40.9287
M. monspessulanus	0.1622	42.0112
M. monspessulanus	0.4566	40.6672
M. monspessulanus	0.5029	42.5594
M. monspessulanus	0.6514	41.8422
M. monspessulanus	0.8921	41.8468
M. monspessulanus	0.9038	41.4868
M. monspessulanus	1.0069	42.0290
M. monspessulanus	1.2606	41.5828
M. monspessulanus	1.3691	42.0347
M. monspessulanus	1.4962	41.7663
M. monspessulanus	1.6087	42.1279
M. monspessulanus	1.8538	41.9505
M. monspessulanus	1.8618	41.5003
M. monspessulanus	1.9830	41.4113
M. monspessulanus	2.2158	41.9536
M. monspessulanus	2.3336	42.2245
M. monspessulanus	2.5789	41.7753
M. monspessulanus	2.6966	42.3161
M. monspessulanus	2.6988	41.8658
M. monspessulanus	2.9396	42.0463
M. monspessulanus	3.4169	43.6501
M. monspessulanus	3.7769	44.0101
M. monspessulanus	4.1768	44.3902
M. monspessulanus	4.4969	43.6501
M. monspessulanus	6.1585	43.1034
M. monspessulanus	6.2970	43.4702
M. monspessulanus	6.6228	43.1765
M. monspessulanus	-1.1014	35.3361
M. monspessulanus	7.1131	35.5215
M. monspessulanus	-8.9764	29.7183
M. monspessulanus	8.4434	35.5675

Table S5 - Summary statistics of Maxent performance, each bioclimatic variable contribution (%) and output (logistic threshold for the "10th percentile", "Equal sensitivity and specificity" and "Maximum sensitivity plus specificity") for models of *H. hippocrepis* and *M. monspessulanus*.

**Metric/Variable**	*H. hippocrepis*	*M. monspessulanus*
N Training / test samples	227/ 96	256/109
Training AUC	0.96 (0.002)	0.94 (0.002)
Test AUC	0.95 (0.004)	0.93 (0.006)
AUC Standard Deviation	0.0043	0.0065
Bio6	1.2 (0.51)	2.3 (2.25)
Bio7	1.1 (0.35)	1.2 (0.58)
Bio8	24.6 (3.11)	30.7 (2.14)
Bio9	14.5 (2.21)	14.2 (3.56)
Bio15	23.8 (3.01)	15.9 (2.51)
Bio16	34.8 (1.49)	35.7 (2.51)
10th percentile	0.39 (0.03)	0.39 (0.02)
Equal sen. And spe.	0.39 (0.02)	0.41 (0.01)
Maximum sen plus spe.	0.26 (0.06)	0.31 (0.04)

Table S6 - Fossil data of *Hemorrhois hippocrepis* (source: fosFARbase, Bohme & Ilg, 2003). Mya: million years ago; epoch boundaries follow Gradstein et al (2005).

Species query	Locality	Country	Latitude	Longitude	Mya range	Epoch
Coluber sp.	Sète, Cap de Lazaret, Hérault	France	43.400	3.683	3.5 - 3.6	Late Pliocene
Coluber sp.	Las Higueruelas, Alcolea de Calatrava, Ciudad Real	Spain	38.988	-4.086	3.1 - 3.3	Late Pliocene
Coluber hippocrepis	Ahl al Oughlam (near Casablanca)	Morocco	33.570	-7.512	2.5 - 2.5	Early Pleistocene
Coluber sp.	Meda Grande, Medas Islands, Catalonia	Spain	42.050	3.200	1.85 - 1.85	Early Pleistocene
Coluber sp.	Grajas Cave, Malaga Province	Spain	36.696	-5.269	0.127 - 0.7	Middle Pleistocene
Coluber sp.	Cova del Rinoceront, eastern Garraf Massif, Can´Aymerich quarry, Castelldelfs	Spain	41.274	1.961	0.074 - 0.147	Late-Middle Pleistocene
Coluber hippocrepis	Cueva del Boquete de Zafarraya, Sierra de Alhama, Málaga	Spain	36.967	-4.133	0.01 - 0.127	Late Pleistocene
Coluber hippocrepis	Cueva Horá (Darro, Granada)	Spain	37.350	-3.300	0.01 - 0.127	Late Pleistocene
Coluber hippocrepis	Higueral de Valleja Cave	Spain	36.750	-5.810	0.021 - 0.057	Late Pleistocene
Coluber hippocrepis	Gorham´s Cave IV, Gibraltar Peninsula	England	36.120	-5.342	0.027 - 0.039	Late Pleistocene
Coluber hippocrepis	Gruta da Figueira Brava, Arrábida	Portugal	38.568	-9.148	0.03 - 0.03	Late Pleistocene
Coluber hippocrepis	Gorham´s Cave IIIb, Gibraltar Peninsula	England	36.120	-5.342	0.017 - 0.023	Late Pleistocene
Coluber hippocrepis	Gorham´s Cave IIIa, Gibraltar Peninsula	England	36.120	-5.342	0.013 - 0.015	Late Pleistocene

Böhme M, Ilg A. fosFARbase. 2003. www.wahrestaerke.com. Accessed March 2021.

Gradstein F, Ogg J, Smith AG. 2005. A Geologic Time Scale 2004. Cambridge University Press, Cambridge, 610 p.

Table S7 - Fossil data of *Malpolon monspessulanus* (source: fosFARbase, Bohme & Ilg, 2003). Mya: million years ago; epoch boundaries follow Gradstein et al (2005).

Species query	Locality	Country	Latitude	Longitude	Mya range	Epoch
Malpolon monspessulanus	Barranco del Cigarrón Puerto de la Cadena northern flank Carrascoy mountain range Murcia	Spain	37.919	-1.161	4.9 - 5	Early Pliocene
Malpolon monspessulanus	Sète Cap de Lazaret Hérault	France	43.400	3.683	3.5 - 3.6	Late Pliocene
Malpolon sp.	Balaruc 2 Massif de la Gardiole near Séte Hérault	France	43.450	3.683	3.1 - 3.3	Late Pliocene
Malpolon sp.	Seynes	France	43.500	3.700	2.7 - 2.9	Late Pliocene
Malpolon monspessulanus	Cova Bonica (CBo) Massif of Garraf Gavà Barcelona	Spain	41.380	1.930	2.5 - 2.6	Early Pleistocene
Malpolon sp.	Ahl al Oughlam (near Casablanca)	Morocco	33.570	-7.512	2.5 - 2.5	Early Pleistocene
Malpolon monspessulanus	Casablanca 4 (ACB-4) Almenara Castellón	Spain	39.750	-0.217	2.3 - 2.35	Early Pleistocene
Malpolon monspessulanus	Cova de La Foradada (CF) Massif de Garraf Barcelone	Spain	41.300	1.900	1.5 - 2.5	Early Pleistocene
Malpolon monspessulanus	Casablanca 1 (ACB-1) Almenara Castellón	Spain	39.750	-0.217	1.8 - 1.9	Early Pleistocene
Malpolon sp.	Casablanca 1 (ACB-1) Almenara Castellón	Spain	39.750	-0.217	1.8 - 1.9	Early Pleistocene
Malpolon monspessulanus	Grotte du Vallonnet	France	43.772	7.475	0.8 - 1.8	Early Pleistocene
Malpolon monspessulanus	Barranco León 5 (BL-5=Capa D) Dépression de Guadix-Baza Grenade	Spain	37.500	-3.000	1.3 - 1.3	Early Pleistocene
Malpolon monspessulanus	El Chaparral Villaluenga del Rosario Cádiz	Spain	36.698	-5.389	0.8 - 1.8	Early Pleistocene
Malpolon monspessulanus	Bagur-2 (B-2) Begur Girone	Spain	41.950	3.217	0.781 - 1.806	Early Pleistocene
Malpolon monspessulanus	Fuente Nueva-3 (FN-3) Dépression de Guadix-Baza Grenade	Spain	37.717	-2.400	1.2 - 1.2	Early Pleistocene
Malpolon monspessulanus	Cueva de la Victoria-1 (CV-1) Carthagène Murcia	Spain	37.617	-0.867	1.1 - 1.2	Early Pleistocene
Malpolon monspessulanus	Cueva de la Victoria-1 (CV-1) Carthagène Murcia	Spain	37.617	-0.867	1.1 - 1.2	Early Pleistocene
Malpolon monspessulanus	Casablanca 3 (ACB-3) Almenara Castellón	Spain	39.750	-0.217	1.1 - 1.1	Early Pleistocene
Malpolon monspessulanus	Quibas-Sima (QS-2 to QS-4) N of Murcia W of Alicante	Spain	38.314	-1.078	1.036 - 1.072	Early Pleistocene
Malpolon monspessulanus	Breches de la Vallette Herault	France	46.667	2.317	0.01 - 1.64	Early-Late Pleistocene
Malpolon monspessulanus	Cova d'Olopte (Cova B) Isòvol Cerdanya	Spain	42.387	1.493	0.126 - 0.781	Middle Pleistocene
Malpolon monspessulanus	Terra Amata Alpes Maritime	France	43.698	7.289	0.127 - 0.7	Middle Pleistocene
Malpolon monspessulanus	Grajas Cave Malaga Province	Spain	36.696	-5.269	0.127 - 0.7	Middle Pleistocene
Malpolon monspessulanus	Cueva del Agua Iznalloz Granada	Spain	36.933	-1.933	0.011 - 0.7	Middle-Late Pleistocene
Malpolon monspessulanus	Lazaret C Alpes Maritimes	France	43.690	7.295	0.12 - 0.2	Middle Pleistocene
Malpolon monspessulanus	Cova del Rinoceront eastern Garraf Massif Can'Aymerich quarry Castelldelfs	Spain	41.274	1.961	0.074 - 0.147	Middle-Late Pleistocene
Malpolon monspessulanus	Valdemino Cave 26-27 (Borgio Verezzi Liguria)	Italy	44.163	12.452	0.1 - 0.11	Late Pleistocene
Malpolon monspessulanus	El Harhoura 1 (Temara)	Morocco	33.950	-6.933	0.01 - 0.2	Middle-Late Pleistocene
Malpolon monspessulanus	Cueva del Camino Secteur Nord Pinilla del Valle Madrid	Spain	40.925	-3.806	0.084 - 0.1	Late Pleistocene
Malpolon monspessulanus	Cueva del Camino Secteur Central Pinilla del Valle Madrid	Spain	40.925	-3.806	0.083 - 0.099	Late Pleistocene

Species query	Locality	Country	Latitude	Longitude	Mya range	Epoch
Malpolon monspessulanus	Hortus Cave Valflaunès Herault	France	43.800	3.875	0.01 - 0.127	Late Pleistocene
Malpolon monspessulanus	Cova del Gegant Massif of Garraf Sitges Barcelona	Spain	41.233	1.733	0.011 - 0.126	Late Pleistocene
Malpolon monspessulanus	Cova del Gegant Massif of Garraf Sitges Barcelona	Spain	41.233	1.733	0.011 - 0.126	Late Pleistocene
Malpolon monspessulanus	Cueva del Boquete de Zafarraya Sierra de Alhama Málaga	Spain	36.967	-4.133	0.01 - 0.127	Late Pleistocene
Malpolon monspessulanus	Higueral de Valleja Cave	Spain	36.750	-5.810	0.021 - 0.057	Late Pleistocene
Malpolon monspessulanus	Teixoneres II Cave	Spain	41.800	1.900	0.03 - 0.038	Late Pleistocene
Malpolon monspessulanus	Gorham's Cave IIIb Gibraltar Peninsula	England	36.120	-5.342	0.017 - 0.023	Late Pleistocene
Malpolon monspessulanus	Gorham's Cave IIIa Gibraltar Peninsula	England	36.120	-5.342	0.013 - 0.015	Late Pleistocene
Malpolon monspessulanus	Valdavara-1 cave Lugo	Spain	42.850	-7.160	0.004 - 0.015	Holocene
Malpolon sp.	Guenfouda Cave (Ghar Zebouj) Jerada Province	Morocco	34.433	-2.000	0.006 - 0.01	Holocene
Malpolon monspessulanus	La Ventana cave UE 14 22 24 26 (neolithic) Torrelaguna Madrid	Spain	40.833	-3.533	0.003 - 0.009	Holocene
Malpolon monspessulanus	El Harhoura 2 (Temara)	Morocco	33.952	-6.926	0.005 - 0.005	Holocene

Böhme M, Ilg A. fosFARbase. 2003. www.wahrestaerke.com. Accessed March 2021.

Gradstein F, Ogg J, Smith AG. 2005. A Geologic Time Scale 2004. Cambridge University Press, Cambridge, 610 p.

Supplementary Figures

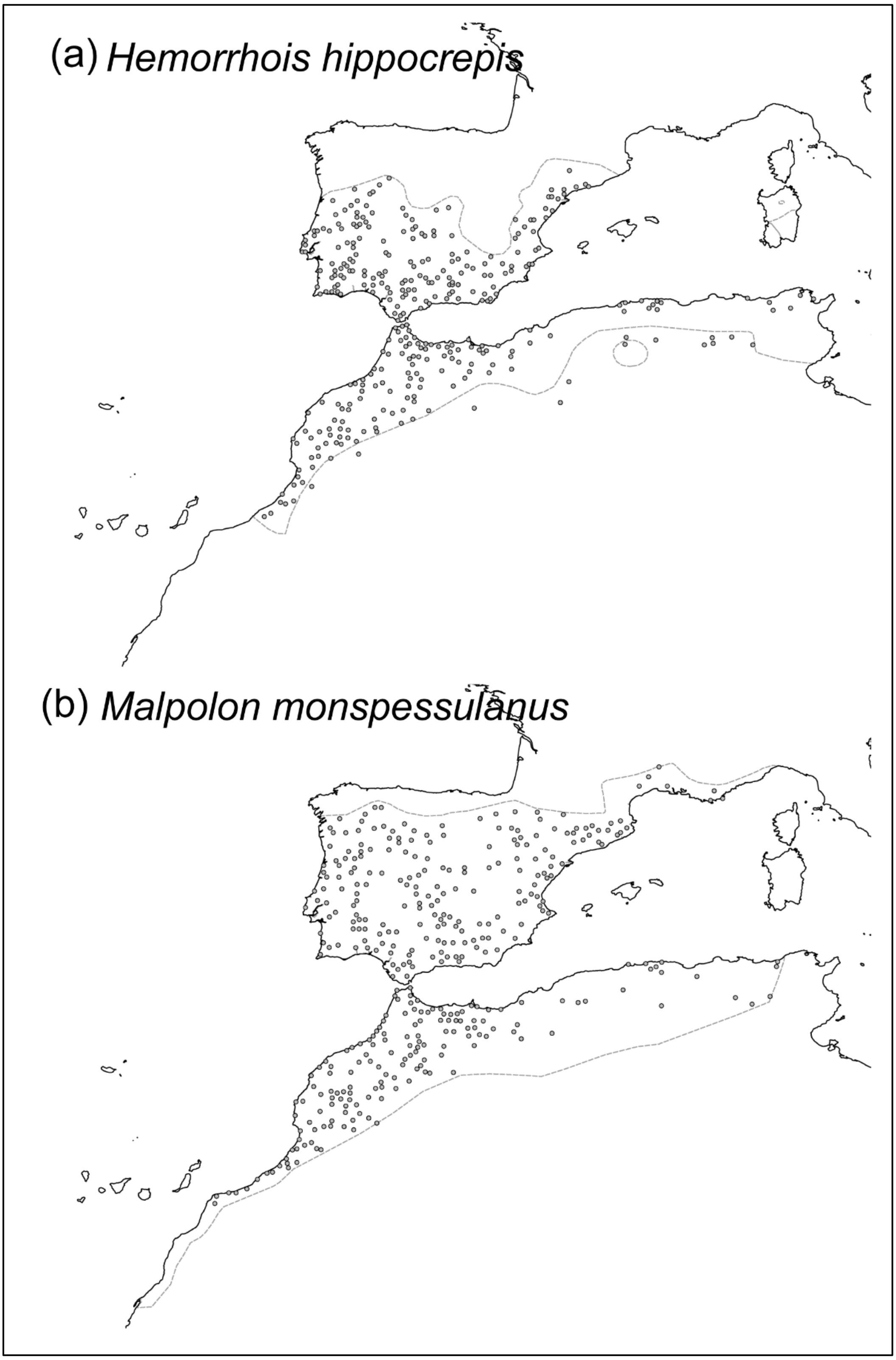

Figure S1: Occurrence points used for Maxent climate modelling: a) *H. hippocrepis*; b) *M. monspessulanus*. Dashed lines represent species native distribution.

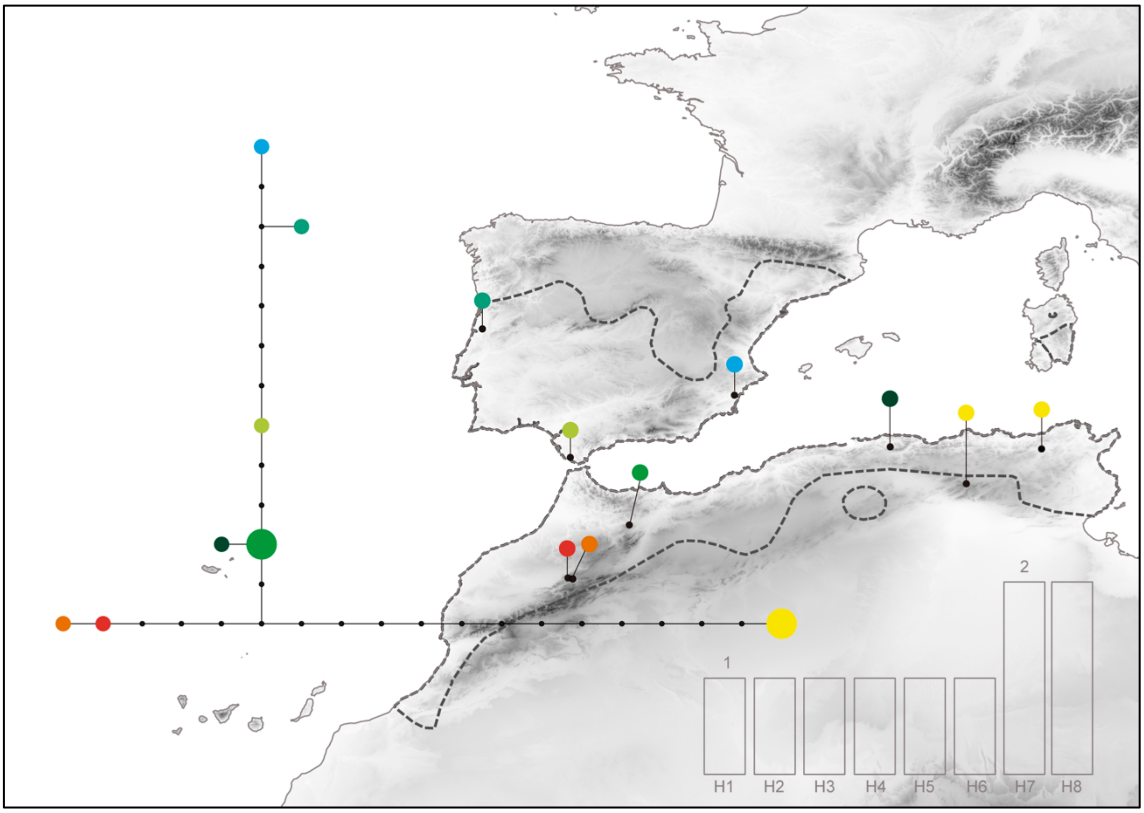

Figure S2: Haplotype network recovered in *H. hippocrepis* using the 700 bp cyt-b fragment.

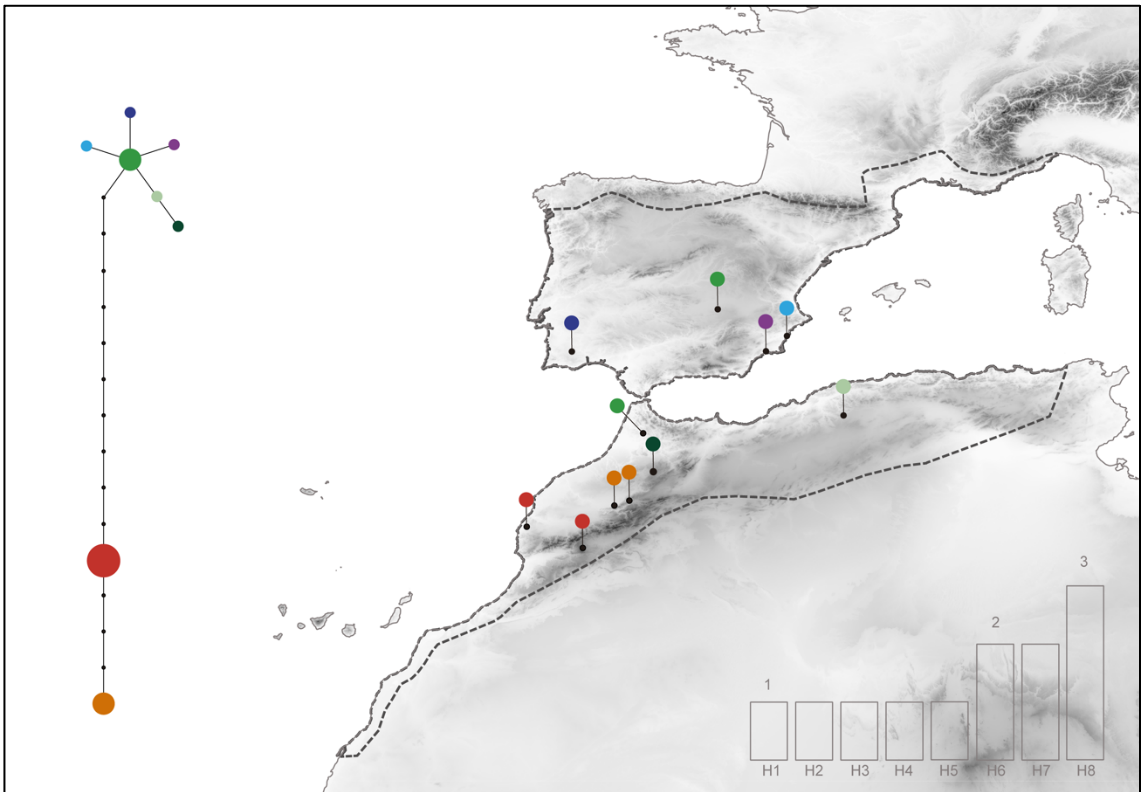

Figure S3: Haplotype network recovered in *M. monspessulanus* using the 700 bp cyt-b fragment.

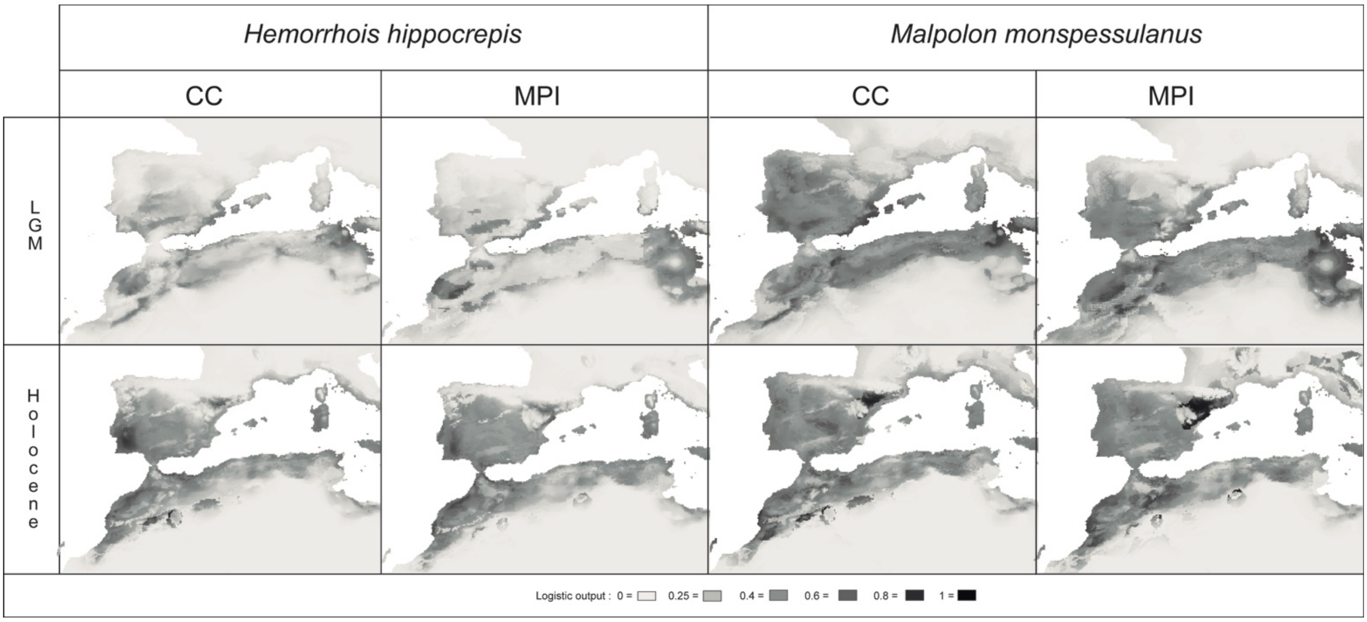

Figure S4: Species distribution models (SDMs) of *H. hippocrepis* and *M. monspessulanus* for Holocene and Last Glacial Maximum (LGM) conditions based on the CC and MPI circulation models.
